# The multiple biological activities of hyperoside: from molecular mechanisms to therapeutic perspectives in neoplastic and non-neoplastic diseases

**DOI:** 10.3389/fphar.2025.1538601

**Published:** 2025-03-03

**Authors:** Weisong Zhang, Rui Wang, Rongqi Guo, Zhongquan Yi, Yihao Wang, Hao Wang, Yangyang Li, Xia Li, Jianxiang Song

**Affiliations:** ^1^ Department of Thoracic Surgery, Affiliated Hospital 6 of Nantong University, Yancheng Third People’s Hospital, Yancheng, China; ^2^ Medical School of Nantong University, Nantong, China; ^3^ Central Laboratory, Affiliated Hospital 6 of Nantong University, Yancheng Third People’s Hospital, Yancheng, China; ^4^ Department of General Medicine, Affiliated Hospital 6 of Nantong University, Yancheng Third People’s Hospital, Yancheng, China

**Keywords:** hyperoside, tumor, antioxidant, anti-inflammatory, signaling pathway, non-neoplastic diseases

## Abstract

In recent years, hyperoside (quercetin 3-O-β-D-galactopyranoside) has garnered significant attention due to its diverse biological effects, which include vasoprotective, antioxidant, anti-inflammatory, and anti-tumor properties. Notably, hyperoside has shown remarkable potential in cancer therapy by targeting multiple mechanisms; it induces apoptosis, inhibits proliferation, blocks angiogenesis, and reduces the metastatic potential of cancer cells. Furthermore, hyperoside enhances the sensitivity of cancer cells to chemotherapy by modulating key signaling pathways. Beyond neoplastic diseases, hyperoside also presents promising therapeutic applications in managing non-cancerous conditions such as diabetes, Alzheimer’s disease, and pulmonary fibrosis. This review comprehensively examines the molecular mechanisms underlying hyperoside’s anti-cancer effects and highlights its role in the treatment of cancers, including lung and colorectal cancers. Additionally, it explores the latest research on hyperoside’s potential in addressing non-neoplastic conditions, such as pulmonary fibrosis, diabetes, and Parkinson’s disease. By summarizing current findings, this review underscores the unique therapeutic value of hyperoside and its potential as a multifunctional treatment in both neoplastic and non-neoplastic contexts.

## 1 Introduction

**FIGURE 1:**
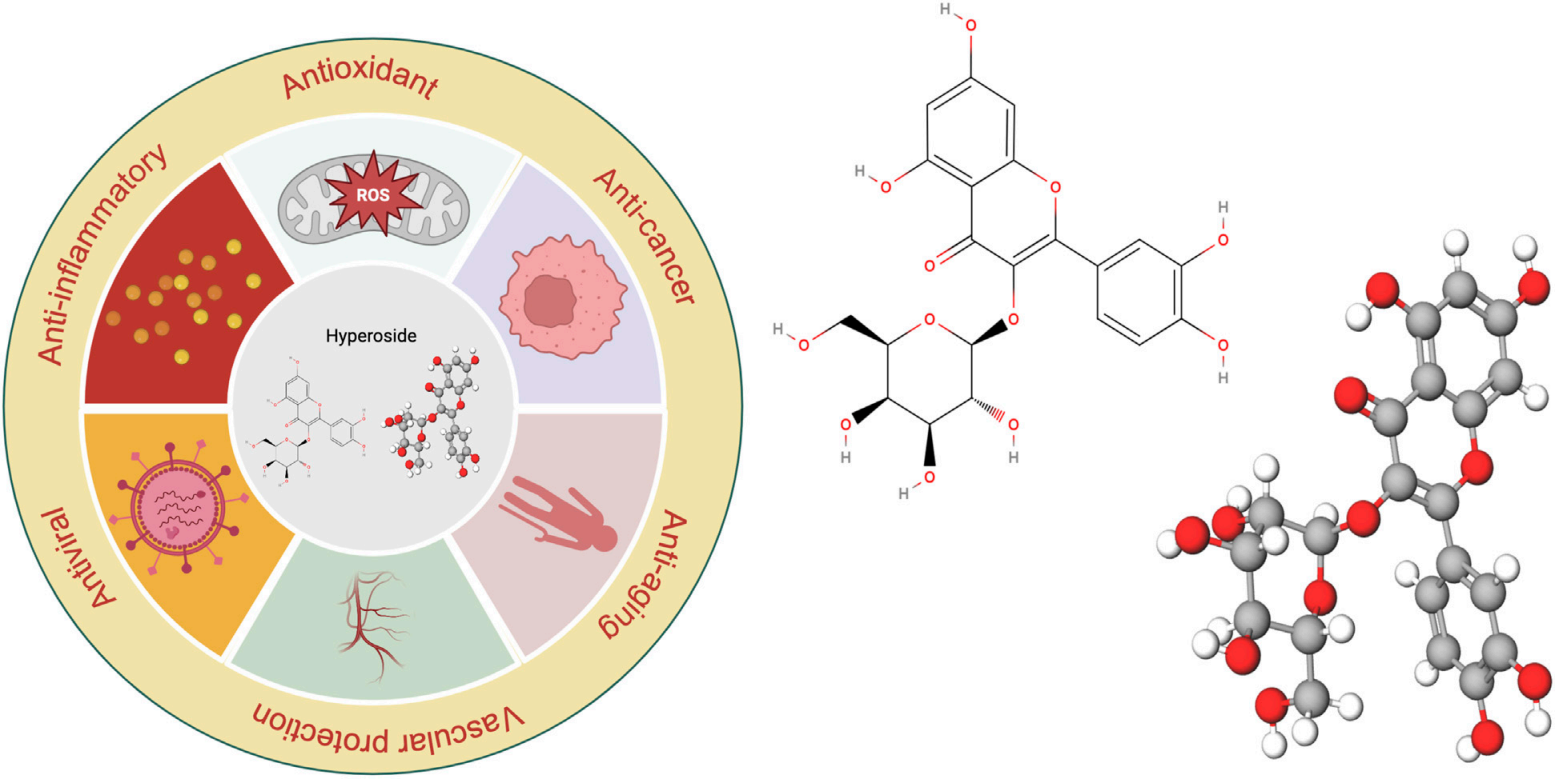
Visualization of the chemical structure and diverse biological activities of hyperoside.

Substances of natural origin play an essential role in the medical field, exhibiting a wide and diverse range of applications. These substances encompass not only natural ingredients derived from plants, animals, and minerals but also traditional herbal remedies and therapies. Among these, hyperoside, a natural compound, has garnered significant attention from researchers due to its diverse biological effects (Figure 1). Hyperoside, a flavonoid compound, is a polyphenolic substance found abundantly in various parts of plants, including flowers, leaves, and fruits. Research indicates that the consumption of flavonoid-rich compounds may confer multiple health benefits (Wen et al., 2021). Hyperoside is commonly found in the fruits and herbs of the Hypericaceae, Rosaceae, Ericaceae, Leguminosae, and Celastraceae families (Orzelska-Górka et al., 2019; Jiang et al., 2021; Karakaya et al., 2022). It is utilized in the treatment of conditions such as high blood pressure and arthritis (Cao et al., 2021). Consequently, hyperoside can be regarded as a common nutrient with properties that include antioxidant, anti-aging, anti-inflammatory, anti-viral, vascular protective, and cancer-preventive effects (Feng et al., 2019; Sun et al., 2021; Wei et al., 2021). The anticancer properties of hyperoside are intricately linked to various biological pathways and their associated mechanisms. As a flavonoid, hyperoside can exert significant effects on cancer cells through multiple mechanisms. It not only inhibits tumor cell proliferation but also induces apoptosis in these cells. Furthermore, hyperoside influences tumor cell growth and migration by modulating relevant signaling pathways, and it enhances the sensitivity of tumor cells to certain chemotherapy drugs. These characteristics highlight hyperoside’s considerable potential in anticancer research.

**FIGURE 2:**
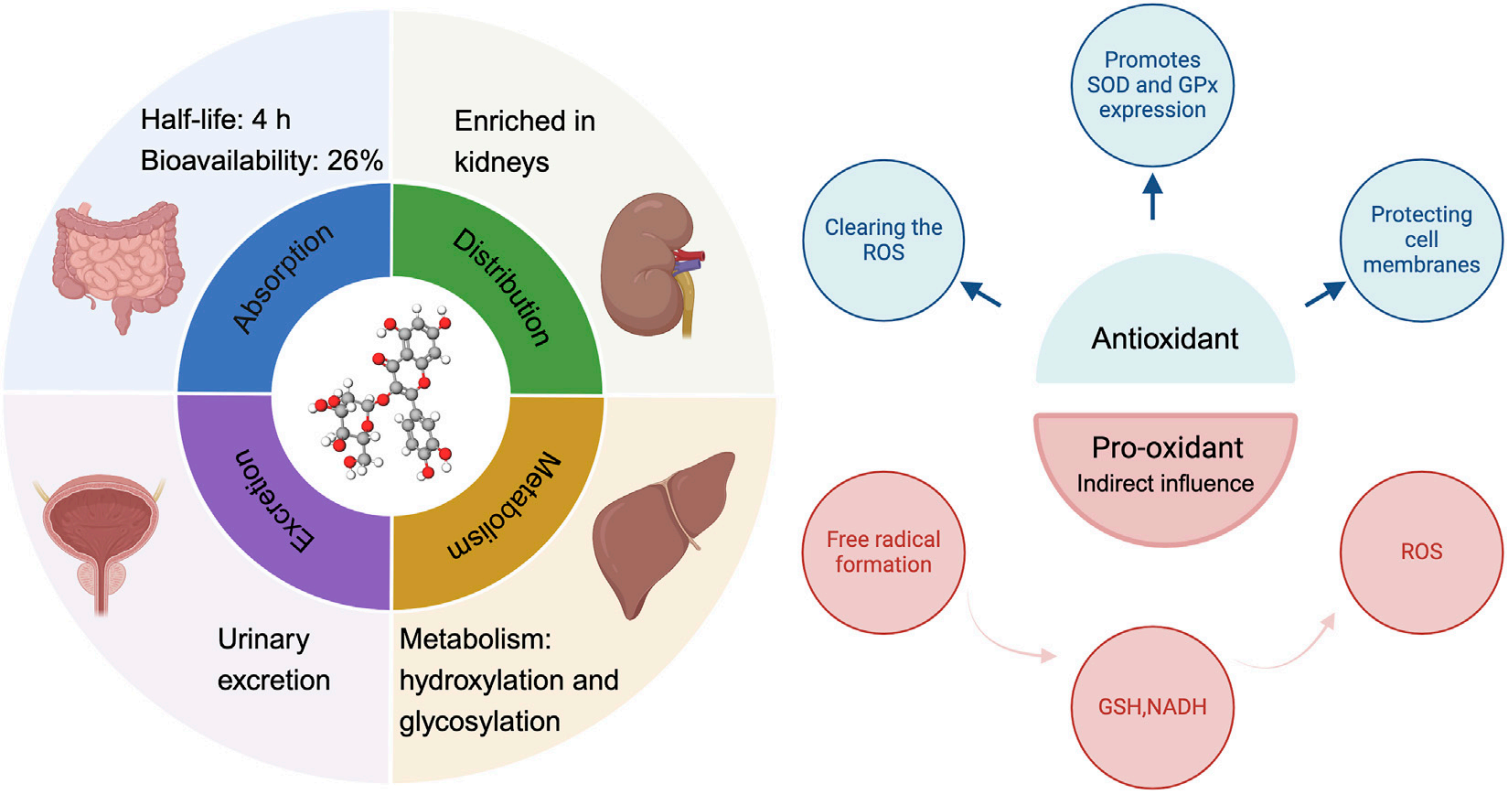
The ADME process of hyperoside and its antioxidant and pro-oxidant properties. ADME (absorption, distribution, metabolism, excretion).

Hyperoside predominantly exists in nature as a glycosylated flavonoid. Specifically, it consists of the natural flavonoid quercetin, which is linked to galactose via a β-glycosidic bond. Its molecular formula is C_21_H_20_O_12_, which corresponds to a molecular weight of 464.3763. The unique chemical structure of hyperoside underpins its diverse biological functions (Ferenczyova et al., 2020). Notably, the multiple hydroxyl functional groups distributed across its A and B rings (with the C ring primarily involved in forming glycosidic bonds) confer potent antioxidant, anti-inflammatory, and anti-tumor effects. These biological activities are mediated through the scavenging of reactive oxygen species (ROS) and the regulation of cell signaling pathways (Wang et al., 2023a). For instance, hyperoside activates the nuclear factor erythroid 2–related factor 2/Heme Oxygenase 1 (Nrf2/HO-1) signaling pathway, neutralizes reactive oxygen species, and inhibits lipid peroxidation, thereby mitigating oxidative stress damage to the heart, liver, and nervous system. These properties underscore the strong correlation between the chemical structure of hyperoside and its biological activity. Previous research indicates that hyperoside is absorbed in the small intestine, with its primary accumulation site being the kidneys. The metabolic process of hyperoside is relatively complex and predominantly occurs in the liver, where the enzyme system significantly influences the metabolism of hyperoside. Common metabolic pathways include hydroxylation and glycosylation. Notably, hyperoside is primarily excreted through urine. While there is currently insufficient direct evidence to demonstrate the impact of renal health on the excretion profile of hyperoside, its accumulation in the kidneys underscores the necessity for further investigation into its potential relationship with renal function. Following intragastric administration of hyperoside to rats, researchers observed a half-life of approximately 4 h and an absolute bioavailability of 26%. This finding suggests that hyperoside could be developed into oral formulations for clinically relevant applications, particularly when combined with compounds such as 2-hydroxypropyl-β-cyclodextrin, a widely utilized drug delivery vehicle. This compounding enhances its bioavailability, which is crucial for its therapeutic efficacy (Shah et al., 2015; Li et al., 2016; Su et al., 2023; Wang et al., 2023a). Similar to other flavonoids, hyperoside acts as an antioxidant, protecting cells by directly scavenging ROS and reducing hydrogen peroxide (H_2_O_2_)-induced lipid peroxidation and protein carbonylation. Furthermore, hyperoside can induce the expression of antioxidant enzymes, such as superoxide dismutase (SOD) and glutathione peroxidase (GPx), thereby enhancing the antioxidant capacity of cells (Piao et al., 2008). ROS are natural by-products of oxygen metabolism, typically maintained at relatively low levels under physiological conditions. However, ROS serve not only as metabolic waste products; they function as “redox messengers” and play crucial roles in cell signaling, cell cycle regulation, gene expression control, and the maintenance of physiological homeostasis. Furthermore, when the body is exposed to stimuli such as ultraviolet light, radiation, hypoxia, or heat, ROS levels can increase dramatically. In these instances, the generation rate of ROS surpasses the body’s scavenging capacity, disrupting the balance between oxidation and antioxidants and leading to oxidative stress. The consequences of oxidative stress are multifaceted, resulting in DNA damage that can lead to mutations and dysfunction of genetic material. Additionally, lipid peroxidation may compromise the integrity of cell membranes, impairing normal cellular functions. Oxidative stress can also induce alterations in the structure and function of proteins, thereby disrupting various biochemical reactions within cells. Ultimately, the accumulation of these damages may lead to cell membrane destruction and potentially cell death, adversely affecting overall health (Maldonado et al., 2020; Huang R. et al., 2021; Zhang Y. et al., 2021; Szarka et al., 2022). The antioxidant activity of hyperoside is attributed to the interaction of multiple molecular mechanisms. This complex structural framework renders it highly effective and reliable in the antioxidant process. In addition to its previously mentioned ability to scavenge ROS and induce antioxidant enzymes, hyperoside can also mitigate the effects of oxidative stress by modulating apoptosis signaling pathways. Its cytoprotective effect is mediated through the activation of the Kelch-like ECH-associated protein 1-Nrf2-Antioxidant Response Element (Keap1-Nrf2-ARE) signaling pathway, which plays a crucial role in cellular defense against oxidative stress (Wang et al., 2023a). Activation of Nrf2 not only enhances the expression of antioxidant genes, thereby effectively reducing oxidative damage, but also downregulates the expression of Bcl-2 and X-linked inhibitor of apoptosis protein (XIAP). This mechanism aids in inhibiting apoptosis, thereby safeguarding cells from oxidative stress-induced damage (Xing et al., 2015). The integration of these mechanisms has led to the identification of hyperoside as an antioxidant, underscoring its multifaceted modes of action. This characteristic suggests the potential clinical value of hyperoside (Figure 2). We also summarized the antioxidant mechanisms and effects of hyperoside across various cell models (Table 1).

**TABLE 1:** Summary of antioxidant mechanisms and effects of hyperoside in different cell models.

Models	Dosage	Mechanisms	Main effects	References
MC3T3-E1 cells	20–40 μmol/L effective; 40 μmol/L optimal	Inhibits the MAPK pathway (p-JNK and p-p38) and regulates the Bcl-2/Bax ratio	Reduces ROS levels, inhibits apoptosis, and improves osteoblast viability	Qi et al. (2020)
Saccharomyces cerevisiae	Effective at 10–40 mg/L	Activates CTT1, SOD1, GSH; reduces ROS and lipid peroxidation	Enhances cell viability, lowers ROS, protects membrane integrity under oxidative stress	Gao et al. (2019)
Human SH-SY5Y cells	Effective at 0.5–2 μM; no cytotoxicity observed in this range	Activates Nrf2-mediated HO-1 pathway; reduces ROS accumulation and mitochondrial dysfunction	Protects neuronal cells, reduces oxidative stress, prevents apoptosis, and restores mitochondrial function	Kwon et al. (2019)
B16F10 melanoma cells	5–50 μM; no cytotoxicity observed in this range	Reduces ROS, ONOO−, •O_2_, and NO• levels; enhances GSH/GSSG ratio and catalase activity	Suppresses melanin synthesis, reduces oxidative stress, and restores redox balance	Jung Kim (2012)
Human L02 hepatocytes	10–800 μM; significant protective effects observed at 200 µM	Activates MAPK–Keap1–Nrf2–ARE pathway; increases HO-1 expression and ROS scavenging	Protects cells, boosts antioxidants, reduces ROS, and prevents mitochondrial dysfunction	Xing et al. (2011)
Mouse B16 melanoma cells	18.2 μM	Suppresses tyrosinase protein expression, reducing melanin biosynthesis	Inhibits melanogenesis and melanin accumulation in a dose-dependent manner	Ohguchi et al. (2010)
ECV-304 human umbilical vein endothelial cells	Effective at 128 µM	Upregulates SIRT1, blocks Bax translocation, reduces cytochrome c release, and restores mitochondria	Prevents apoptosis, reduces lipid peroxidation, DNA damage, and restores redox balance	Li et al. (2008)
V79-4 lung fibroblast cells	Effective at 5 μM; no cytotoxicity observed	Scavenges ROS, enhances antioxidant enzymes, reduces lipid peroxidation and DNA damage	Protects cells, inhibits apoptosis, restores antioxidant balance	Piao et al. (2008)
PC12 cells	10–160 μg/mL; optimal protection observed at 100–160 μg/mL	Scavenges ROS, reduces LDH release, inhibits apoptosis, stabilizes mitochondria	Protects cells, enhances viability, decreases apoptosis, restores balance	Liu et al. (2005)

Surprisingly, while the antioxidant capacity of flavonoids has long been recognized and valued, recent research has increasingly demonstrated that these compounds may also exhibit pro-oxidant properties (Eren-Guzelgun et al., 2018). Flavonoids can self-oxidize in the presence of transition metals, leading to the generation of superoxide anions. This process is particularly significant in their metabolism, especially due to the presence of phenolic rings, which enable flavonoids to form pro-oxidative free radicals. The generation of these free radicals not only plays a crucial role in metabolic processes but also participates in co-oxidation reactions with glutathione (GSH) and nicotinamide adenine dinucleotide (NADH), thereby influencing the redox state within cells. Furthermore, NADH, an important energy transfer molecule, is closely associated with ROS. Consequently, the free radicals produced during the autooxidation and metabolism of flavonoids may have a significant and profound impact on ROS formation (Pérez-Torres et al., 2017; Mohammadi et al., 2024). Hyperoside demonstrates dual characteristics in modulating the redox state, a duality influenced by various factors, including the concentration of hyperoside and the specific characteristics of the cellular environment (Zhang et al., 2017; Feng et al., 2021b). If the biological activity of hyperoside in specific cellular microenvironments can be accurately predicted, it may demonstrate significant potential for clinical applications. Hyperoside is a compound known for its antioxidant properties, which effectively safeguard cells from damage induced by oxidative stress. Given the strong correlation between oxidative stress and cellular mutations, this protective effect positions hyperoside as a promising agent in the prevention of tumor transformation (Park et al., 2016).

Hyperoside has demonstrated anticancer activity and is effective against various types of cancer, including lung cancer, colorectal cancer, bladder cancer, pancreatic cancer, and hepatocellular carcinoma (Fu et al., 2016; Hu et al., 2019; Qiu et al., 2019; Wang et al., 2023a; Yang et al., 2024). Studies indicate that it not only inhibits cancer progression *in vitro* but also suppresses tumor cell proliferation *in vivo*. Furthermore, this flavonoid compound can disrupt the cell cycle, thereby inhibiting tumor growth and inducing apoptosis. The relationship between hyperoside and tumor-related diseases is complex and will be explored in greater depth in this article. The compound’s significant anti-cancer properties suggest its potential utility in cancer prevention and treatment. Additionally, hyperoside can modulate inflammatory processes, which is particularly relevant given the role of inflammation in the development of many diseases. It reduces the release of inflammatory factors by influencing various signaling pathways, including the Toll-like Receptor 4 (TLR4) signaling pathway induced by lipopolysaccharide (LPS), as well as the Wnt/β-catenin and Sonic Hedgehog signaling pathways (Jin et al., 2016; Huang J. et al., 2021; Yang et al., 2022). Research has shown that this compound significantly inhibits the production of key pro-inflammatory factors, such as tumor necrosis factor (TNF-α), interleukin-6 (IL-6), and nitric oxide (NO) (Kim et al., 2011). Moreover, hyperoside can promote the production of anti-inflammatory factors, such as IL-10, thereby achieving anti-inflammatory effects (Tang et al., 2023). This bidirectional regulatory property suggests that hyperoside may have significant potential for application in the treatment of inflammation. In addition to the areas of pulmonary fibrosis and diabetes discussed in this review, a recent article on hyperoside highlights its analgesic properties, which may be associated with its anti-inflammatory effects (Rahman et al., 2023).

It is important to note that while hyperoside demonstrates promising therapeutic properties and shows potential application value in medical research, it’s possible toxicity and potential health effects must be carefully considered prior to practical application (Xu S. et al., 2022). Relevant studies indicate that flavonoids may interact with certain medications, potentially leading to either increased or decreased drug efficacy. For instance, specific flavonoids may influence the liver’s role in drug metabolism, which can result in heightened drug toxicity or diminished therapeutic efficacy (Galati and O’Brien, 2004; Zong et al., 2024). Additionally, research indicates that unborn fetuses are particularly sensitive to flavonoids, as these compounds can easily cross the placental barrier and may impact fetal development. Furthermore, other studies have shown that flavonoids exhibit only slight cytotoxicity to normal cells at high concentrations. This finding not only underscores the relative safety of flavonoids at elevated concentrations but also provides a crucial foundation for future application research (Vanhees et al., 2011). Available studies indicate that hyperoside exhibits relatively low acute toxicity. No adverse effects were observed in short-term animal studies at doses up to 5,000 mg/kg. However, long-term exposure may be associated with potential teratogenic effects, such as reduced fetal growth, highlighting the necessity for dose optimization and more rigorous toxicological studies. Future research should prioritize chronic toxicity assessments, organ-specific toxicity investigations, and special attention to safety in vulnerable populations, including pregnant women and children. Additionally, hyperoside may influence cytochrome P450 enzyme activity, which could pose a potential risk for drug metabolism during combination therapy; thus, detailed drug interaction studies are essential to mitigate associated risks. Furthermore, structural modifications to hyperoside could be pursued to enhance its safety while preserving its therapeutic efficacy. Risk assessment strategies that incorporate advanced toxicological modeling, computer simulations, and *in vivo* experiments will be critical for transitioning from preclinical studies to clinical applications. By proactively addressing these safety concerns, hyperoside is expected to be developed into a safer and more effective multipurpose therapeutic agent.

**FIGURE 3:**
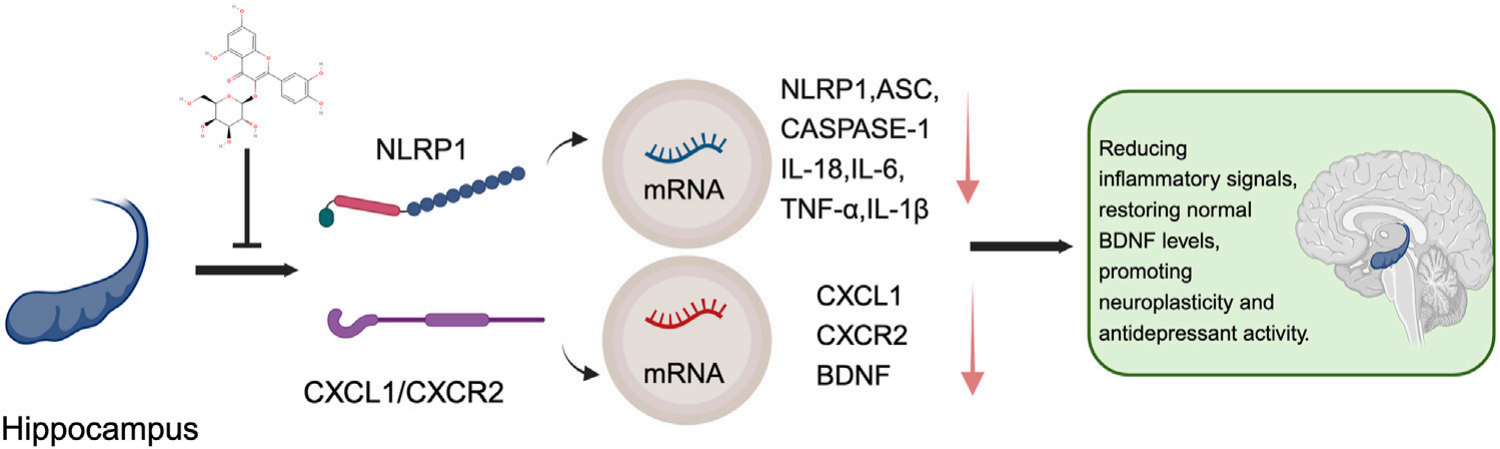
Schematic visualization of the antidepressant mechanism of hyperoside.

Due to their natural origin, flavonoids are currently widely utilized as dietary supplements, particularly for alleviating anxiety and enhancing mental health. Additionally, hyperoside, a type of flavonoid, possesses antioxidant properties, making it suitable for use in cosmetics and skincare products aimed at combating skin aging and improving skin tone. This further underscores the safety and potential applications of hyperoside (Mirabile et al., 2023). Relevant research indicates that hyperoside is regarded as having high safety. For instance, in a 14-day acute toxicity experiment, mice administered doses up to 666 times the effective dose of hyperoside (5,000 mg/kg) exhibited no significant abnormalities in behavioral activities, blood biochemical indicators, or other scientific metrics. This finding suggests that the acute toxicity of hyperoside is relatively low. Specifically, the oral median lethal dose (LD50) in mice exceeds 5,000 mg/kg, which corresponds to an approximate human dose of 549.5 mg/kg (Ai and Huang, 2012). This data indicates that the safety of hyperoside in mice has been effectively validated under acute exposure conditions, suggesting that it may be better tolerated in humans when administered in appropriate amounts. Furthermore, the research team employed the bacterial reverse mutation test (Ames test) and chromosomal aberration test to assess the genotoxic effects of the tested compounds. The results demonstrated that hyperoside did not induce mutations in various strains (including TA97, TA98, TA900, and TA102), and there were no significant alterations in the chromosomal structure of CHL fibroblasts. This finding implies that hyperoside is not genotoxic (Ai and Huang, 2012). An experimental study on rat embryo and fetal development revealed that at a dosage of 1,000 mg/kg, the compound had minimal effects on pregnant rats; however, it did lead to a reduction in the growth rate of fetal rats (Ai et al., 2012b). It is important to note that long-term use of hyperoside may result in certain toxic effects on the kidneys, although this damage is reversible (Ai et al., 2012a). Hyperoside is the primary active component of *Hypericum perforatum* (HP), a medicinal plant that has been utilized for centuries and is widely recognized as an effective treatment for mild to moderate depression. HP is often considered the only herbal alternative to traditional synthetic antidepressants, highlighting its significance and unique value in the management of depression (Oliveira et al., 2016; Radulović et al., 2018). Currently, tricyclic antidepressants and Selective Serotonin Reuptake Inhibitor(s) (SSRIs) reuptake inhibitors are the most widely used antidepressants (Bighelli et al., 2018). However, despite the high prevalence of depression, significant shortcomings remain in the effectiveness and safety of depression treatments globally. Chinese herbal medicine, as a potential source of antidepressant therapy, may offer considerable benefits to patients suffering from depression (Fathinezhad et al., 2019). Relevant studies have demonstrated that hyperoside exerts antidepressant effects by activating the Extracellular Signal-Regulated Kinase-Brain-Derived Neurotrophic Factor (ERK-BDNF) signaling pathway, which involves extracellular signal-regulated kinase (Zheng et al., 2012). Furthermore, hyperoside was assessed for its antidepressant activity in the forced swim test (FST) for the first time (Butterweck et al., 2000). Research has established a correlation between BDNF levels and the efficacy of antidepressants (Gelle et al., 2021). Recent studies revealed that the expression levels of CXCL1 and its receptor CXCR2 were significantly upregulated in a chronic unpredictable mild stress (CUMS)-induced depression-like mouse model. The study further indicated that overexpression of CXCL1 in the hippocampus not only triggers the emergence of depressive-like behaviors but also results in a reduction of BDNF levels. Inhibiting the activity of CXCR2 helps prevent depressive-like behaviors and restore normal BDNF levels (Chai et al., 2019). Additionally, the NOD-like Receptor Protein 1(NLRP1) inflammasome influences the onset of depressive-like behaviors induced by chronic stress by regulating the C-X-C Motif Chemokine Ligand 1/Receptor 2- Brain-Derived Neurotrophic Factor (CXCL1/CXCR2-BDNF) signaling pathway in mice (Song et al., 2020; Zhu Y.-J. et al., 2022). A recent study utilized UPLC-QTOF-MS/MS technology to investigate the antidepressant effects of hyperoside, the primary active ingredient in HP. Through network pharmacology, it was found that hyperoside exhibits multi-target and multi-component synergistic effects, thereby exerting its therapeutic impact. To validate this correlation, the researchers conducted *in vivo* experiments that demonstrated hyperoside’s significant efficacy in improving depressive-like behavior in mice subjected to chronic stress. In the CUMS model, the mice displayed pronounced depressive-like behaviors, characterized by slowed body weight gain, significantly prolonged immobility times in both the FST and the tail suspension test (TST), as well as markedly reduced preference rates in the sucrose preference test. In comparison to the CUMS group, mice treated with hyperoside and paroxetine exhibited a significant reduction in immobility times in the FST and TST, alongside a notable improvement in the sucrose preference rate. This evidence strongly indicates that hyperoside effectively alleviates depression-like symptoms induced by chronic stress. Regarding the exploration of its molecular mechanisms, hyperoside significantly decreased the expression of inflammasome-related molecules NLRP1, ASC, and caspase-1 in the hippocampus of CUMS group mice, while also inhibiting the mRNA levels of pro-inflammatory cytokines such as IL-18, IL-6, TNF-α, and IL-1β. These findings suggest that hyperoside mitigates the inflammatory response associated with chronic stress by inhibiting the activation of the NLRP1 inflammasome. Hyperoside also modulated the mRNA expression of stress-related CXCL1 and CXCR2 in the hippocampus, significantly reducing their levels while simultaneously up-regulating the expression of BDNF. The increase in BDNF is closely associated with antidepressant effects. Consequently, the authors concluded that hyperoside is the primary antidepressant component in *H. perforatum* (HP), and its antidepressant effect may ameliorate depressive-like behavior in mice through the NLRP1 signaling pathway (Figure 3). Furthermore, these effects may be mediated by the CXCL1/CXCR2/BDNF signaling pathway (Song et al., 2022). Flavonoids are prevalent in the plant kingdom, originating from natural and healthy sources, and are commonly found in everyday dietary components such as fruits and vegetables. Due to their diverse pharmacological activities, including antioxidant, anti-inflammatory, and neuroprotective properties, hyperoside has demonstrated significant potential for development as a dietary supplement and a prospective intervention for mental health (Xia et al., 2024). Hyperoside exhibits distinct pharmacological characteristics within the flavonoid family, setting itself apart through its dual redox modulation capacity, specificity in signaling pathway regulation, broad therapeutic potential across various diseases, and superior safety and pharmacokinetic profiles. Its targeted actions on pathways such as TLR4, Nrf2-ARE, and CXCL1/CXCR2, combined with its dual antioxidant and pro-oxidant properties, position it as a prime candidate for the development of multi-target therapeutic agents. Consequently, a comprehensive investigation into hyperoside’s mechanisms of action and clinical application potential not only supports its advancement in the treatment of both neoplastic and non-neoplastic diseases but also establishes a critical scientific foundation for the development of functional foods and pharmaceutical innovations.

## 2 Hyperoside: modulation of carcinogenesis

Hyperoside demonstrates significant potential for intervention in the pathogenesis of various cancers. The formation of cancer is a complex, multi-stage process that involves several key mechanisms, including clonal expansion, cell proliferation, and metastasis. To enhance our understanding of hyperoside’s role in tumorigenesis, we provide a concise overview of the critical stages of carcinogenesis. Initially, cancer development is often initiated by exogenous or endogenous carcinogens. Through the action of metabolic enzymes, these cancer-promoting agents are converted into mutagenic compounds, resulting in DNA damage characterized by various types of genetic mutations, such as base substitutions, insertions, or deletions. These mutations compromise normal cellular control mechanisms, leading to uncontrolled cell proliferation and disruption of the normal cell life cycle. Subsequently, cells enter a cancer-promoting phase, during which genetically damaged cells continue to proliferate, thereby accelerating the irreversible initiation of tumors. This phase indicates that the cells not only retain the capacity for continued proliferation but also acquire invasiveness toward surrounding tissues while successfully evading immune system surveillance. Following this, in the tumor progression stage, the malignancy of cancer cells significantly escalates, and their invasiveness and metastatic capabilities are markedly enhanced. Furthermore, these cancer cells secure the necessary nutritional support by promoting angiogenesis, which facilitates the formation of new blood vessels, thereby further accelerating tumor expansion. At this stage, tumor cells exhibit the ability to proliferate rapidly throughout the body, driven by increased angiogenesis, and they display a series of unique properties closely associated with alterations in their cell signaling pathways (Fishbein et al., 2020; Fishbein et al., 2021). Hyperoside possesses the capacity to modulate significant biological pathways by interacting with multiple targets. In the subsequent sections, we will elaborate on the most critical of these signaling pathways.

## 3 Hyperoside: modulation of cell proliferation

Hyperoside can activate or inhibit the cell proliferation process through various mechanisms. It regulates cell proliferation by either inhibiting cell cycle progression or by suppressing tumor epidermal growth factor receptor (EGFR)-mediated signaling pathways associated with cell proliferation. Flavonoids have demonstrated significant potential in inhibiting tumorigenesis, primarily through the regulation of the cell cycle. These compounds effectively prevent the progression of the cell cycle at the G1/S and G2/M checkpoints, thereby impacting cell proliferation and growth. Specifically, flavonoids inhibit the advancement of cells at these two critical checkpoints, resulting in the inability of tumor cells to divide and proliferate efficiently, which reduces the rate of tumor development (Mancini et al., 2021; Pai et al., 2021; Xi et al., 2022). Hyperoside plays a pivotal role in regulating the cell cycle, particularly through its ability to induce cell arrest in the G1/S phase. This phase is crucial for cell growth and division and is often considered a key regulatory point in the cell’s life cycle. When cells are influenced by hyperoside, their processes are effectively halted, particularly during the DNA synthesis stage, thereby inhibiting the proliferation of tumor cells. Notably, this finding has been corroborated in various types of tumor cells, including those from lung cancer, bladder cancer, hepatocellular carcinoma, and osteosarcoma (Zhang et al., 2014; Liu et al., 2016; Li et al., 2018; Wei et al., 2021; Yang et al., 2024). A study demonstrated that hyperoside exerts an inhibitory effect on the lung cancer cell line A549, with its mechanism involving the suppression of cyclin D1 (CCND1) and cyclin-dependent kinase 4/6 (CDK4/CDK6) expression. Both proteins are integral to the G1/S transition of the cell cycle. Consequently, the effect of hyperoside indicates its potential to inhibit the proliferation of lung cancer cells by inducing cell cycle arrest in the G1 phase. Furthermore, hyperoside has been shown to promote apoptosis in lung cancer cells in a dose-dependent manner. Interestingly, this study also investigated whether hyperoside exhibits a synergistic effect with let-7a-5p from the let-7 microRNA family in inhibiting A549 cell proliferation. MicroRNAs (miRNAs), which are non-coding RNA molecules ranging from 17 to 23 nucleotides in length, play a crucial role in gene expression regulation. They influence the expression of post-transcriptional genes by binding to the 3′ untranslated region, 5′ untranslated region, or coding sequence of target genes (Valencia-Sanchez et al., 2006; Bartel, 2009). In the context of human cancer pathogenesis, miRNAs are recognized to play a pivotal role. Acting as tumor suppressors, miRNAs significantly impact the initiation and progression of cancer by regulating genes associated with tumor development. Notably, the let-7 miRNA family is widely acknowledged as a tumor suppressor in various human tumors (Ma et al., 2010; Nadiminty et al., 2012b; Nadiminty et al., 2012a). Hyperoside synergizes with let-7a-5p to significantly inhibit cell proliferation by inducing a G1/S phase block. This effect is mediated by the downregulation of both mRNA and protein levels of CCND1, a mechanism that has been validated using qRT-PCR and Western blot techniques. Notably, the combination of Hyperoside and let-7a-5p demonstrates significantly greater efficacy in reducing CCND1 expression and inhibiting cell proliferation compared to either agent alone. This synergistic effect underscores the therapeutic potential of Hyperoside in enhancing the tumor suppressor function of microRNAs. Additionally, Hyperoside has been shown to promote apoptosis through mitochondrial pathways by modulating key apoptosis-related proteins, such as Bcl-2, Bax, and caspase-3. Although the interaction between Hyperoside and let-7a-5p in promoting apoptosis has not been directly explored, their combined efficacy in inhibiting cell proliferation suggests the potential for an enhanced pro-apoptotic effect. This synergistic interaction not only targets tumor cell proliferation but also highlights a promising strategy to overcome resistance mechanisms commonly associated with single-agent therapies (Li et al., 2018). Based on these findings, the interaction between Hyperoside and non-coding RNAs, such as let-7a-5p, presents a novel direction for cancer therapy. The ability of Hyperoside to modulate the activity of non-coding RNAs and enhance their tumor suppressor functions holds significant promise for cancer treatment. Future studies should focus on further investigating the molecular mechanisms underlying this synergistic effect, particularly its potential implications for apoptosis induction and its interaction with other non-coding RNAs across various cancer types. Furthermore, a study in colon cancer revealed that hyperoside treatment modulated the expression of apoptosis-related markers, including Bax, cleaved caspase-3, and cleaved caspase-7. Researchers utilized flow cytometry to assess cell cycle arrest and apoptosis in the human colorectal cancer cell line SW620 treated with hyperoside, finding that hyperoside may inhibit cell growth by disrupting the G2/M phase of the cell cycle. Furthermore, the study revealed that the reduction in Bax expression correlated with a concurrent decrease in p53 expression. Simultaneously, hyperoside treatment resulted in the upregulation of expression levels of cytochrome c, Apaf-1, caspase-9, and caspase-3, with significant differences observed when compared to the control group. These findings suggest that p53 and caspase-dependent signaling pathways are crucial in hyperoside-induced apoptosis (Zhang et al., 2017).

**FIGURE 4:**
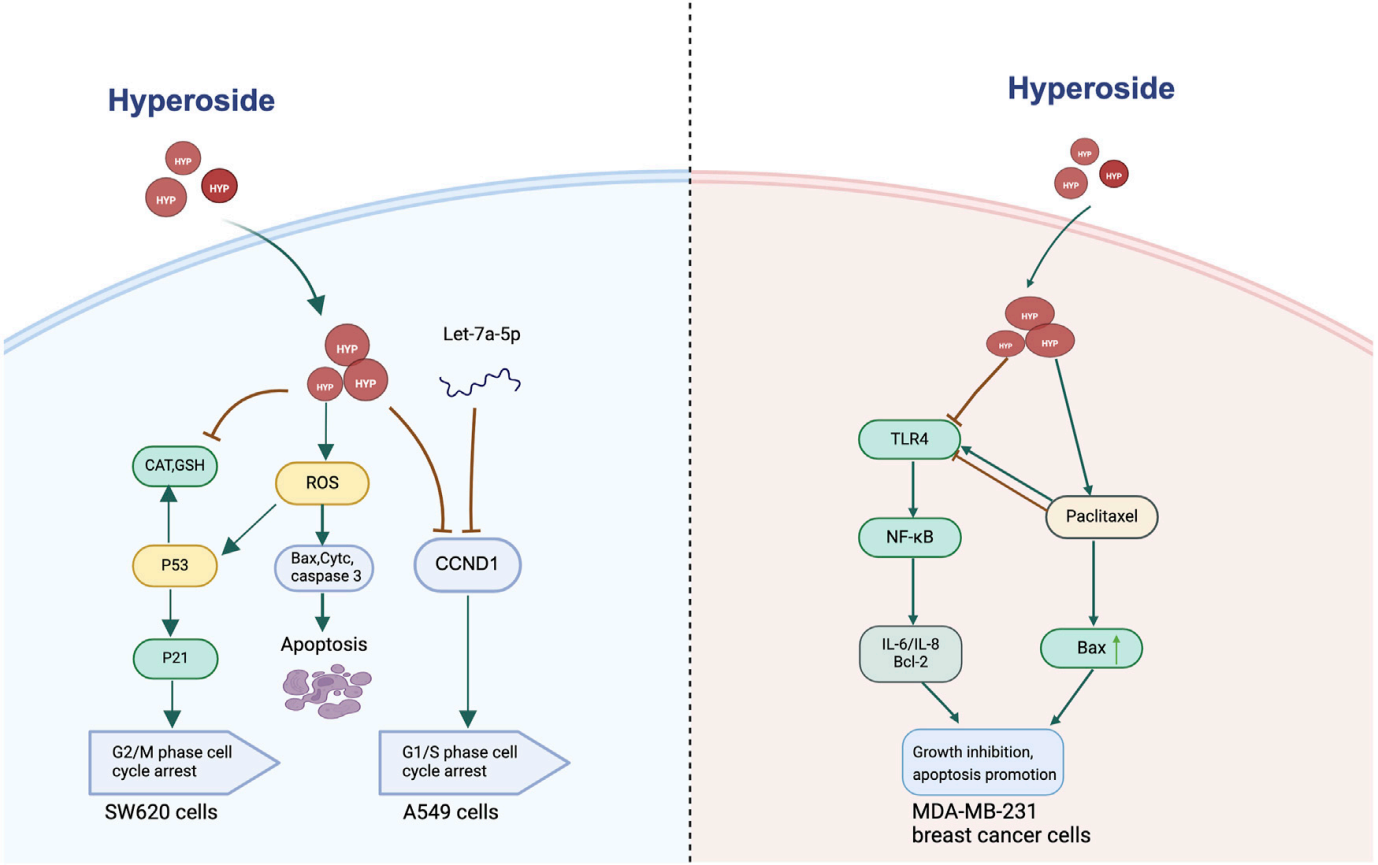
Hyperoside-mediated cell cycle regulation and enhancement of paclitaxel chemosensitivity.

Research has demonstrated that hyperoside can influence the efficacy of chemotherapy agents. Specifically, hyperoside modulates the anti-tumor activity of drugs by regulating the interaction pathways between these agents and cancer cells. Notably, hyperoside exhibits significant synergistic effects in increasing the sensitivity of breast cancer cells to the chemotherapy drug paclitaxel. This enhancement is primarily achieved through the inhibition of the TLR4 signaling pathway (Figure 4). Although paclitaxel is a widely utilized anti-cancer drug, it frequently encounters the issue of drug resistance in the treatment of advanced breast cancer (Volk-Draper et al., 2014; Wang C.-H. et al., 2018). This resistance is partly attributed to paclitaxel’s ability to promote the expression of pro-inflammatory mediators and anti-apoptotic proteins via the activation of the TLR4-NF-κB pathway (Szajnik et al., 2009; Sootichote et al., 2018). Evidence suggests that hyperoside effectively obstructs this signaling pathway, leading to a reduction in TLR4 expression and subsequent NF-κB activation, which in turn inhibits tumor cell proliferation. Specifically, hyperoside not only decreases the expression of anti-apoptotic proteins such as Bcl-2 but also enhances the activation of the pro-apoptotic marker Bax by paclitaxel. Furthermore, hyperoside reduces the levels of pro-inflammatory cytokines, including IL-6 and IL-8, thereby diminishing the survival capacity of tumor cells induced by paclitaxel. Importantly, this synergistic effect is particularly pronounced in TLR4-positive MDA-MB-231 breast cancer cells, while the effect is less evident in HCC1806 cells that lack TLR4, underscoring the critical role of TLR4 in the therapeutic combination of hyperoside and paclitaxel. Additionally, the study found that hyperoside enhanced the sensitivity of paclitaxel in breast cancer cells while exhibiting minimal toxicity to normal breast cells. This conclusion was supported by experiments conducted on normal human breast epithelial cells, including the MCF-10A cell line. The results indicated that hyperoside effectively inhibited the proliferation of breast cancer cells, such as the MDA-MB-231 cell line, while demonstrating almost no significant impact on the proliferation and viability of normal breast cells (Sun et al., 2020).

Hyperoside exhibits significant dual potential, effectively inhibiting cell proliferation signaling mediated by the EGFR, thereby underscoring its crucial role in modulating cell growth. Furthermore, this compound interferes with key components across multiple signaling pathways, which enhances its anti-proliferative properties by targeting pathways associated with cell survival, such as MAPK and NF-κB. The interplay of these mechanisms enables hyperoside to exert substantial effects on regulating cell proliferation and survival. Consequently, hyperoside can be regarded as not only an effective inhibitor of cell proliferation but also a promoter of cell apoptosis, indicating its promising applications in cancer treatment and related fields. Growth factors, including platelet-derived growth factor (PDGF), epidermal growth factor (EGF), insulin-like growth factor (IGF), and TNF-α, are critical bioactive molecules that play a vital role in promoting cancer cell proliferation. These factors activate intracellular signaling pathways by binding to specific proteins on the cell membrane known as growth factor receptors, which are essential for regulating cell growth and differentiation. Notable growth factor receptors include the EGFR and the insulin-like growth factor receptor (IGFR). Abnormal activation of these receptors is closely associated with the onset and progression of various cancers (Da Cunha Santos et al., 2011; Dickerson et al., 2024). Research results on hyperoside indicate that it can significantly interfere with multiple signaling pathways and effectively inhibit the proliferation of cancer cells. In specific experiments, the study confirmed its mechanism of action through Western blot technology. The results demonstrated that hyperoside can significantly inhibit the activity of key signaling pathways, such as mitogen-activated protein kinase (MAPK), mammalian target of rapamycin (mTOR), and Protein Kinase B (PKB/Akt), thereby reducing the expression of the EGFR (Yang et al., 2024). Notably, overexpression of EGFR often promotes the proliferation of cancer cells. Thus, hyperoside effectively inhibits cancer cell proliferation by lowering EGFR levels. Although studies have shown that hyperoside has a substantial inhibitory effect on cell proliferation, these investigations are primarily based on *in vitro* cell models, which may not fully capture the more complex environmental factors present in living organisms. Consequently, to more comprehensively and thoroughly evaluate the antiproliferative potential of hyperoside, additional animal experiments and related clinical studies are urgently needed.

## 4 Hyperoside: induction of apoptosis

Apoptosis refers to the programmed self-destruction of cells, a process essential for maintaining cellular balance and normal functions within an organism. This mechanism is significant for several reasons: it plays a crucial role in the development of organisms, immune responses, and tissue homeostasis. Dysregulation of apoptosis is closely associated with the onset of numerous diseases, particularly cancer. By eliminating cells that are no longer necessary or may pose a threat—such as those with irreversible DNA damage—apoptosis effectively preserves the stability of a cell population. The apoptotic process is tightly regulated and is primarily categorized into two pathways: intrinsic and extrinsic. The intrinsic pathway is activated by internal stress signals, such as DNA damage or oxidative stress, while the extrinsic pathway is initiated by external signals, such as cytokines (Hanahan and Weinberg, 2011; Morana et al., 2022). Functional defects within the apoptotic pathways, particularly abnormalities in death receptors or mitochondrial pathways, are often linked to tumorigenesis. Although both the mitochondrial and death receptor pathways can elicit similar apoptotic outcomes, their origins and molecular mechanisms exhibit significant differences.

**FIGURE 5:**
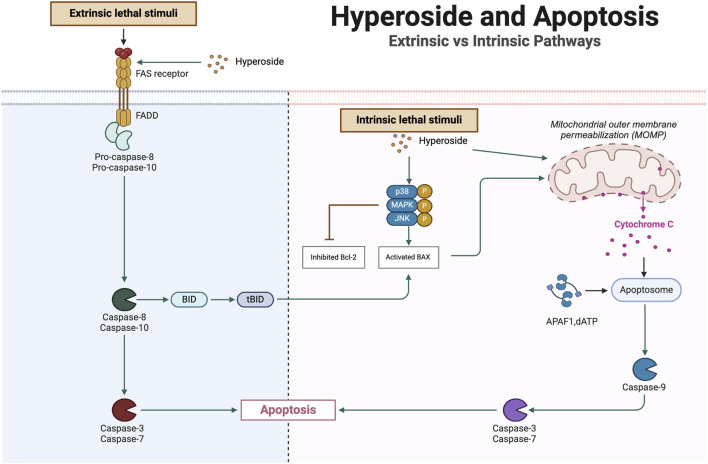
Hyperoside-induced apoptosis: mitochondrial and death receptor pathways.

In the mitochondrial pathway, the expression levels of Bcl-2 family members, such as Bax and Bik, increase, which promotes the release of cytochrome c. The released cytochrome c activates caspase-9, subsequently activating effector caspases, including caspase-3 and caspase-7, ultimately leading to the degradation of intracellular proteins. In the death receptor pathway, cytokines from the tumor necrosis factor (TNF) family, such as TNF-α and FasL, bind to specific death receptors, forming the death-inducing signaling complex (DISC). This complex comprises the linker molecule Fas-Associated Death Domain (FADD) and the precursor of caspase-8. Activated caspase-8 cleaves the pro-apoptotic protein Bid, resulting in the generation of the tBid fragment, which translocates to the mitochondrial membrane and triggers the release of pro-apoptotic substances, including cytochrome c (Pradelli et al., 2010; Van Herreweghe et al., 2010; Fuchs and Steller, 2011; Kaufmann et al., 2012; Cavalcante et al., 2019). Hyperoside promotes the death of cancer cells by enhancing apoptosis mechanisms and diminishing cancer cell survival signals. In the process of inducing apoptosis, hyperoside not only activates mitochondria-related apoptotic pathways but also stimulates death receptor-related signaling pathways (Figure 5). These two mechanisms collaboratively enhance its anti-tumor effect. Studies have demonstrated that flavonoids can induce DNA damage through mechanisms such as the inhibition of DNA topoisomerase activity and the promotion of p53 protein activation (Goodenow et al., 2020). Additionally, hyperoside has been shown to significantly enhance the phosphorylation levels of p38 MAPK and c-Jun N-terminal kinase (JNK). These alterations lead to the disruption of mitochondrial membrane permeability, facilitating the release of cytochrome c and apoptosis-inducing factors from the mitochondria into the cytoplasm (Yang et al., 2017c). Such processes are pivotal in triggering the apoptosis cascade. Furthermore, hyperoside induces cell apoptosis by modulating the expression of Bcl-2 and Bax proteins. Specifically, Bcl-2 functions as an apoptosis inhibitor, whereas Bax promotes apoptosis. Hyperoside enhances Bax expression while inhibiting Bcl-2 expression, thereby increasing the cleavage and activation of caspases. This shift in the expression ratio results in heightened mitochondrial membrane permeability, which further propagates intracellular apoptosis signals and ultimately initiates the apoptotic process (Lü, 2016). In pharyngeal squamous cell carcinoma cell lines (FaDu), hyperoside can induce cell apoptosis by activating the Fas signaling pathway. As a critical death receptor, Fas initiates the downstream caspase cascade reaction upon binding to its ligand. Research indicates that hyperoside upregulates the expression level of Fas, thereby triggering apoptosis through the activation of caspase-8, ultimately leading to the apoptotic pathway mediated by death receptors (Kang et al., 2024). Additionally, hyperoside not only activates apoptosis-related pathways but also inhibits cell survival signaling. Specifically, it suppresses the activity of the NF-κB survival pathway, which plays a significant role in enhancing the apoptotic effect induced by TNF-α, thereby inhibiting tumor progression to some extent (Li et al., 2016; Kang et al., 2024). Notably, a recent study explored the potential of hyperoside to alleviate cardiomyocyte apoptosis induced by doxorubicin (DOX). The findings suggest that hyperoside may effectively inhibit DOX-induced HL-1 cell apoptosis by blocking the activation of the apoptosis signal-regulated kinase 1 (ASK1)/p38 signaling pathway (Chen et al., 2023). This underscores the multidimensional application potential of hyperoside in various cellular contexts. While hyperoside has demonstrated some efficacy in inducing apoptosis, future studies should focus on *in vivo* validation to confirm its apoptotic effects. In particular, clinical trials are necessary to assess its safety and effectiveness in human subjects.

## 5 Hyperoside: inhibition of carcinogen activation

Flavonoids play a significant role in inhibiting the activation of carcinogens. In the human body, carcinogens are often metabolized into active forms that bind to DNA, leading to mutations and cellular damage. Certain flavonoids, such as hyperoside, may exhibit an inhibitory effect on this activation process. Although research has demonstrated that flavonoids, including hyperoside, can significantly inhibit the activity of cytochrome P450 family 1(CYP1) enzymes, it should be noted that these findings predominantly pertain to flavonoids as a collective group rather than hyperoside in isolation. As substrates for the CYP1 enzyme, flavonoids may also facilitate the production of additional metabolites, which possess antiproliferative properties against cancer cells. Furthermore, flavonoids can bind to carcinogens, thereby obstructing their interaction with DNA, which reduces the risk of mutations and mitigates cellular damage caused by these carcinogens. Nevertheless, the specific biological role of hyperoside in these mechanisms requires further investigation (Kale et al., 2008; Androutsopoulos et al., 2010; Martinez-Perez et al., 2014; Wilsher et al., 2017).

## 6 Hyperoside: actions in autophagy

Autophagy is a self-regulated degradation process utilized by cells to adapt to adverse environments. It achieves this by degrading and recycling damaged organelles, proteins, and other cellular components, thereby maintaining cellular homeostasis. This mechanism is crucial in both normal physiological conditions and pathological states, playing a significant role in metabolic regulation, anti-infection responses, and tissue homeostasis. Research has demonstrated that dysregulation of autophagy is closely associated with a variety of diseases, particularly cancer. Autophagy can both promote the survival of tumor cells and inhibit tumor progression by inducing cell death (Galluzzi et al., 2017; Galluzzi et al., 2018).

### 6.1 Anti-tumor effects

Hyperoside, a natural flavonoid with diverse biological activities, may exert varying effects on cell survival and apoptosis across different cell types and pathological conditions by modulating autophagy-related signaling pathways. During the initiation of autophagy, the cytosolic form of LC3-I is converted to the membrane-bound form LC3-II, which associates with phagophores and autophagosomes. This conversion is crucial for the formation of autophagosomes, making LC3-II a widely used marker for assessing autophagosome abundance and autophagy levels (Mizushima and Yoshimori, 2007; Jiang and Mizushima, 2015; Schaaf et al., 2016). A distinctive feature of tumor cells is their highly active metabolism, which is accompanied by oxidative stress and rapid cell proliferation. These characteristics often lead to dysregulation of autophagy, thereby supporting cell survival and growth. In the NSCLC cell line A549, hyperoside promotes autophagy by effectively inhibiting the PI3K/Akt/mTOR signaling pathway, thereby exerting its anti-tumor effects. The PI3K/Akt/mTOR pathway is essential for regulating both cell proliferation and autophagy. The mTOR influences autophagy by modulating downstream effector molecules such as p70S6 kinase (p70S6K) and 4E-BP1 (Hoeffer and Klann, 2010; Zoncu et al., 2011; Laplante and Sabatini, 2012). Following hyperoside treatment, the phosphorylation levels of PI3K and Akt in A549 cells were significantly diminished, which further inhibited mTOR activity and led to a reduction in the phosphorylation levels of its downstream targets, p70S6K and 4E-BP1. This inhibition resulted in a marked increase in the conversion of LC3-I to LC3-II, suggesting that autophagosome formation was enhanced. Concurrently, the expression of p62/SQSTM1, an autophagy substrate, was decreased, further indicating an augmentation of autophagic flux. Flow cytometry and Western blot analyses corroborated the mechanism by which hyperoside induces autophagy via the PI3K/Akt/mTOR pathway. To ascertain whether hyperoside leads to the accumulation of LC3-II by inhibiting autophagic degradation, the autophagy inhibitors E64d and pepstatin A were employed in the experiment. The observed increase in LC3-II levels further supports the conclusion that hyperoside induces cytotoxicity by promoting autophagosome formation. Additionally, experimental results combined with the autophagy inhibitor 3-MA revealed that hyperoside-induced A549 cell death was significantly diminished following 3-MA pretreatment. This finding underscores the critical role of autophagy in facilitating cell death within the anti-tumor mechanism of hyperoside (Fu et al., 2016).

Researchers have demonstrated that hyperoside can induce apoptosis in ovarian cancer cell lines HO-8910 and SKOV3 through the progesterone receptor membrane component 1 (PGRMC1)-dependent autophagy pathway, while also enhancing the cells’ sensitivity to the chemotherapy drug cisplatin. Additionally, studies reveal that PGRMC1 is overexpressed in ovarian cancer. This protein stabilizes the EGFR signaling pathway via its interaction with the cytochrome P450 complex, thereby promoting cancer cell proliferation and enhancing their anti-apoptotic capabilities (Ahmed et al., 2010; Szczesna-Skorupa and Kemper, 2011; Kabe et al., 2016). The conversion of Microtubule-Associated Protein 1 Light Chain 3B-I (LC3B-I) to Microtubule-Associated Protein 1 Light Chain 3B-II (LC3B-II) is recognized as a critical step in autophagosome formation, serving as a reflection of autophagic activity. Variations in LC3B-II levels can effectively indicate the biogenesis and degradation rates of autophagosomes (Li et al., 2020; Peña-Martinez et al., 2022; Hwang and Kim, 2023). Following hyperoside treatment, the levels of LC3B-II in ovarian cancer cells from both groups were significantly elevated, accompanied by an increase in autophagosome formation. Immunofluorescence analysis revealed an enhanced colocalization between LC3B and PGRMC1, suggesting that PGRMC1 plays a crucial role in the autophagy process induced by hyperoside. Further experimental findings indicated that the overexpression of PGRMC1 markedly augmented the induction of autophagy and apoptosis by hyperoside, whereas the knockdown of PGRMC1 inhibited these processes. Western blot analysis demonstrated that the expression levels of autophagy-related proteins LC3B-II and Beclin-1 increased in HO-8910 and SKOV3 cells treated with hyperoside. Conversely, the expression of the autophagy substrate p62 protein decreased, indicating an enhancement in autophagy flux. Additionally, hyperoside disrupts the cell’s anti-apoptotic mechanisms by inhibiting the expression of the Bcl-2 protein downstream of the Akt signaling pathway, thereby promoting cell apoptosis. Flow cytometry results showed that in the hyperoside-treated group, levels of activated caspase-3 and caspase-9 were significantly elevated, further confirming the occurrence of cell apoptosis. In drug combination experiments, hyperoside and cisplatin were co-administered to treat HO-8910 and SKOV3 cells. The results indicated a significant decrease in cell viability in the combined treatment group, along with a notable increase in the proportion of apoptotic cells. This suggests that hyperoside significantly enhances the cytotoxicity of cisplatin through PGRMC1-dependent autophagy regulation (Zhu et al., 2017).

### 6.2 Protective effect

Under normal physiological conditions, healthy cells maintain a balanced autophagic flux. However, in pathological states or under stress, the autophagy process may become excessive or dysregulated. Research indicates that hyperoside can effectively inhibit this aberrant autophagic response, thereby safeguarding cellular health. In the lung injury model induced by particulate matter (PM2.5), hyperoside significantly mitigated lung cell damage by modulating the AMPK/mTOR pathway. Research findings indicate that hyperoside effectively promotes AMPK activation while markedly inhibiting the phosphorylation of mTOR, thereby alleviating the autophagy dysregulation caused by PM2.5 exposure. Such exposure often results in excessive autophagy activity within cells, exacerbating cellular damage (Deng et al., 2013; He et al., 2017). However, hyperoside aids in restoring autophagy homeostasis by downregulating the expression of autophagy-related markers p62 and LC3-II, consequently reducing the rate of cell apoptosis. This study systematically verifies the protective effects of hyperoside through both *in vitro* and *in vivo* experiments. In the *in vitro* studies, organic solvent soluble PMs (O-PMs) significantly heightened autophagy and apoptosis in human bronchial epithelial cells (Beas-2b). Cells pretreated with hyperoside exhibited notable improvements, particularly reflected in the significant downregulation of autophagy-related markers such as beclin-1, atg3, and LC3-II. Furthermore, the expression of apoptosis-related proteins PARP, Bax, and caspase-3 was inhibited, while the level of the anti-apoptotic protein Bcl-2 increased. Research has established that PM2.5-induced cellular damage is closely linked to dysregulation of autophagy mediated by the AMPK/mTOR pathway (Deng et al., 2013). AMPK, an energy-sensing enzyme, typically initiates autophagy by inhibiting the mTOR signaling pathway upon activation (Long et al., 2019; Wu Y.-F. et al., 2020). However, in PM2.5-treated Beas-2b cells, the expression of p-AMPK was significantly elevated, resulting in the overexpression of autophagy-related proteins and subsequent cellular damage. The mechanism by which hyperoside exerts its effects involves the restoration of mTOR activity through the inhibition of AMPK phosphorylation, leading to a reduction in the expression of autophagy-related proteins and a significant decrease in cell damage. In in vivo experiments, mice exposed to PM2.5 exhibited marked structural damage to lung tissue and infiltration of inflammatory cells. Compared to the control group, the concentrations of inflammatory factors such as IL-6 and TNF-α in the bronchoalveolar lavage fluid (BALF) of the PM2.5-treated group were significantly elevated. Furthermore, histological examination via HE staining revealed severe damage to the alveolar walls, accompanied by a substantial accumulation of cells surrounding the lung airways. Following hyperoside pretreatment, there was a notable reduction in both the number of inflammatory cells and the secretion of inflammatory factors in BALF, thereby effectively mitigating lung damage. Western blot analysis indicated a decrease in the level of p-AMPK in the hyperoside-treated group, while the expression of p-mTOR was increased, further supporting the notion that hyperoside exerts a protective effect on the lungs by inhibiting abnormal autophagy mediated by the AMPK/mTOR signaling pathway (Gao et al., 2021).

A recent study has demonstrated that hyperoside may mitigate kidney aging and damage by inhibiting the autophagy mechanism. In a model of renal aging and injury induced by D-galactose (D-gal), hyperoside exhibited significant renal protective effects. The underlying mechanism appears to be associated with the inhibition of the AMPK-ULK1 signaling pathway, which reduces cell damage resulting from autophagy dysregulation. *In vitro* experimental results indicated that hyperoside treatment significantly inhibited the expression of aging markers p53 and p21, while also markedly reducing the levels of pro-inflammatory factors IL-1 and TGF-β. Further analysis revealed that hyperoside downregulates the expression of autophagy-related proteins LC3 and Beclin1, concurrently decreasing the accumulation of autophagosomes, thereby suggesting its potential protective role against D-gal-induced autophagy hyperactivity. Additionally, hyperoside pretreatment significantly inhibited the abnormal increase in markers such as the autophagy-related proteins LC3 and Beclin1. Immunohistochemical analysis further indicated that hyperoside could reduce the positive staining area of LC3 in renal tissue, thereby confirming its role in inhibiting the accumulation of autophagosomes. Hyperoside appears to reduce the activation of Unc-51 Like Autophagy Activating Kinase 1(ULK1) by inhibiting the phosphorylation of AMPK, which in turn diminishes cell damage caused by autophagy. Ultrastructural observations revealed that the number of autophagosomes in renal tubular cells was significantly reduced following hyperoside treatment. The verification experiments involving the AMPK activator 5-Aminoimidazole-4-carboxamide-1-β-D-ribofuranoside (AICAR) and the inhibitor Compound C further confirmed that hyperoside’s role in regulating autophagy primarily depends on the AMPK-ULK1 pathway. Overall, hyperoside effectively inhibited the autophagic process and mitigated D-gal-induced autophagic damage by modulating the AMPK-ULK1 signaling pathway. This finding offers a novel research direction for exploring the application of hyperoside in aging-related diseases (Liu et al., 2024).

### 6.3 Others

In addition to its significant anti-tumor and protective effects, hyperoside demonstrates potential applications across various fields (Table 2).

**TABLE 2:** Mechanisms of hyperoside-mediated autophagy regulation in various pathological models.

Pathological model	Cell/animal model	Type of action	Key signaling pathway	Key molecules and changes	Mechanism description	Final effect
NSCLC	A549 cells	Anti-tumor effect	PI3K/Akt/mTOR	↓PI3K, ↓Akt, ↓mTOR ↑LC3-II, ↓p62	Inhibits PI3K/Akt/mTOR pathway, enhances autophagic flux, and promotes apoptosis	Induces autophagy-mediated apoptosis, inhibits proliferation
Ovarian cancer	HO-8910, SKOV3 cells	Anti-tumor effect	PGRMC1-dependent autophagy	↑PGRMC1 ↑LC3-II, ↑Beclin-1, ↓p62	PGRMC1 enhances autophagy flux and apoptosis pathways	Improves cisplatin chemosensitivity, induces apoptosis
Lung injury (PM2.5 exposure)	Beas-2b cells; mouse model	Protective effect	AMPK/mTOR	↑AMPK, ↓mTOR ↓LC3-II, ↓Beclin-1, ↓p62	Restores autophagy balance and reduces inflammation	Reduces apoptosis and restores lung homeostasis
Kidney injury (D-gal induced)	Renal tubular cells; mouse model	Anti-aging effect	AMPK-ULK1	↓AMPK, ↓ULK1 ↓LC3, ↓Beclin-1	Inhibits excessive autophagy and aging markers	Reduces cell senescence and tissue damage
Cardiac protection (TAC)	TAC-induced HF rats; AngII-induced H9C2 cells	Cardiac protective effect	Autophagy-apoptosis crosstalk	↑LC3-II, ↑Beclin-1, ↓p62 ↑Bcl-2, ↓Bax, ↓Caspase-3	Enhances autophagy flux and suppresses apoptosis	Improves cardiac function and reduces myocardial apoptosis
Neuroprotection (Epilepsy model)	KA-induced epilepsy mouse model	Neuroprotective effect	PI3K/AKT and MAPK	↑PI3K, ↑MAPK ↑SOD1, ↑SOD2 ↓LC3-II, ↓Beclin-1	Balances oxidative stress and autophagy, reduces neuronal damage	Improves neural survival and reduces hippocampal injury

For instance, in the context of heart failure, studies indicate that hyperoside promotes autophagy while inhibiting apoptosis in cardiomyocytes. In a study involving thoracic aortic coarctation (TAC) in rats, the experimental group treated with hyperoside exhibited improved cardiac function and reduced cardiomyocyte apoptosis, a phenomenon linked to enhanced autophagy. When 3-methyladenine (3-MA) was employed to inhibit autophagy, the cardioprotective effects of hyperoside were diminished, further substantiating that its protective mechanism operates through the induction of autophagy (Guo et al., 2020). Additionally, some studies suggest that hyperoside mitigates neuronal damage by regulating autophagy. In a mouse model of epilepsy, hyperoside pretreatment was correlated with increased antioxidant levels and decreased expression of autophagy-related proteins, implying that hyperoside may exert neurological effects by maintaining the balance between autophagy and oxidative stress (Cao et al., 2020).

In summary, the relationship between hyperoside and autophagy is complex and multifaceted, with hyperoside serving as a potential modulator of autophagy across various pathological conditions, including heart failure, cancer, and neurodegenerative diseases. Hyperoside’s capacity to induce autophagy and promote apoptosis indicates that it may represent a valuable therapeutic agent in contexts where autophagy significantly influences disease progression and treatment response. Consequently, it is of considerable scientific and clinical importance to conduct further research aimed at elucidating the specific mechanisms by which hyperoside affects autophagy and its potential implications for therapeutic applications.

The relationship between autophagy and apoptosis is a central factor in maintaining homeostasis of the intracellular environment and influencing the development of disease. Studies have shown that Hyperoside can not only effectively activate apoptosis, but also regulate the autophagy process, demonstrating its potential application in a variety of therapeutic areas. The ability of this compound to induce apoptosis in cancer cells while protecting normal cells by modulating the autophagy mechanism is a good example of targeted therapy and personalized medicine. Future studies should focus on relevant experimental and clinical trials *in vivo* to evaluate the safety and efficacy of hyperoside and their mechanisms of action in various diseases. If hyperoside are incorporated into existing therapeutic strategies, especially in combination with other therapies, it will be possible to open new avenues for precision medicine and find new solutions for diseases that cannot be effectively treated with conventional therapies.

## 7 Hyperoside: actions in lung cancer

Lung cancer has long been recognized as one of the leading causes of death associated with malignant tumors (Mazzone et al., 2016; Siegel et al., 2024). Due to the absence of obvious symptoms in its early stages and the limited accuracy of existing diagnostic technologies, most patients are diagnosed at an advanced stage, which exacerbates the high mortality rate from lung cancer in both men and women. Lung cancer is primarily categorized into small cell lung cancer (SCLC) and non-small cell lung cancer (NSCLC), with NSCLC exhibiting a higher incidence rate (Gridelli et al., 2015). Current treatment modalities for lung cancer include chemotherapy, targeted therapy, and immunotherapy; however, the effectiveness of these treatments is frequently constrained by factors such as cancer type, stage, number of metastases, and the timing of diagnosis (Nooreldeen and Bach, 2021). The benefit of early diagnosis lies in its potential to expand treatment options for patients and significantly enhance survival rates (Thai et al., 2021). Unfortunately, the lack of early symptoms often results in many patients not receiving a diagnosis until the disease has progressed to an advanced stage, which directly impacts treatment efficacy. Although chemotherapy remains a prevalent treatment approach for lung cancer, it is often accompanied by adverse side effects, and patients may develop multidrug resistance (Jänne et al., 2015). Therefore, it is crucial to investigate new diagnostic techniques and treatment alternatives, including the exploration of natural anticancer compounds from plants, such as hyperoside, which may offer promising alternative treatment options.

Immune checkpoints are immunosuppressive molecules primarily expressed on the surface of immune cells. Their function is to regulate the immune system and prevent the onset of autoimmunity. During the tumor immune response, tumor cells can exploit immune checkpoints to inhibit T cell-mediated immune attacks (Andrews et al., 2019; Zhang T. et al., 2021). Proteins such as Cytotoxic T-Lymphocyte Antigen 4(CTLA-4) and PD-L1 can impede T cell immune responses and modulate the mechanisms of immune recognition and evasion to some extent. The Programmed Cell Death Protein 1(PD-1) is predominantly found on the surface of αβ T cells and B cells, where it binds to the PD-L1 and PD-L2 ligands present on tumor cells, consequently inhibiting the activation of immune cells. By blocking PD-L1 on tumor cells and PD-1 on T cells, immunotherapy can effectively restore T cell function and enhance the immune system’s ability to recognize and eliminate tumor cells, thereby achieving anti-tumor effects (Chen et al., 2022; Ren et al., 2022).

Bioactive ingredients found in traditional Chinese medicine, including saponins, terpenoids, alkaloids, and flavonoids, have demonstrated the potential to modulate the expression of programmed death ligand 1 (PD-L1). This regulatory process involves a variety of complex signaling pathways, such as PI3K/Akt/mTOR, P38, NF-κB, and JAK/STAT3. The intervention in these signaling pathways provides diverse mechanisms through which bioactive components can influence immune cell function. By down-regulating PD-L1 expression, these ingredients effectively inhibit the immune evasion of tumor cells, thereby enhancing the immune system’s ability to identify and eliminate these malignant cells. Furthermore, these bioactive ingredients can activate immune cells and enhance their anti-tumor effects, ultimately improving the body’s immune response to tumors (Qu et al., 2018; Jiang et al., 2020; Dong et al., 2021; Jing et al., 2021; Qiu et al., 2021). The anti-tumor effect of hyperoside on NSCLC is primarily manifested through its ability to downregulate the expression of PD-L1. PD-L1 serves as a crucial immune checkpoint protein that inhibits T cell activity by binding to the PD-1 receptor on T cells, thereby facilitating the immune evasion of tumor cells (Zhang T. et al., 2021). Research indicates that hyperoside can significantly reduce the PD-L1 protein levels in NSCLC cell lines H1975 and HCC827 in a concentration- and time-dependent manner. The underlying mechanism of this effect is attributed to the inhibitory action of hyperoside on the transcription factor c-Myc, which plays a pivotal role as an upregulating factor influencing PD-L1 expression. Validation through qRT-PCR and Western blot experiments further confirmed the c-Myc-dependent regulation of PD-L1 by hyperoside at the transcriptional level. Additionally, hyperoside can enhance the cytotoxic effect of Jurkat T cells on NSCLC cells. This suggests that hyperoside not only downregulates PD-L1 expression but also improves the tumor microenvironment, thereby providing significant support for the enhancement of the tumor immune response. In in vivo experiments, the research team utilized the Lewis lung cancer mouse model to conduct a thorough investigation into the efficacy of hyperoside. Following intraperitoneal injection of 25 mg/kg hyperoside, a significant reduction in tumor volume was observed in the mice, resulting in a tumor inhibition rate of 48.3%, with no apparent toxic reactions noted. Immunohistochemical analysis revealed that hyperoside treatment significantly decreased the expression of PD-L1 in tumor tissues, while the number of cells positive for the apoptosis marker cleaved-caspase 3 markedly increased. This further supports the mechanism by which hyperoside exerts its anti-tumor effects through the downregulation of PD-L1 (Dong et al., 2021). In summary, hyperoside effectively inhibits the expression of PD-L1 and c-Myc, successfully counteracting the immune evasion associated with NSCLC and enhancing T cell-mediated cytotoxic responses. These findings suggest that hyperoside holds potential application value in the immunotherapy of non-small cell lung cancer.

The anti-lung cancer properties of hyperoside are closely associated with multiple molecular mechanisms. Firstly, hyperoside effectively inhibits the migration and invasion of A549 lung cancer cells by down-regulating the expression of metastasis-related genes, including MTA1, matrix metalloproteinase-2 (MMP-2), and its inhibitor TIMP-2 (Yang et al., 2017b). Additionally, hyperoside significantly activates the phosphorylation of p38 MAPK and JNK, resulting in damage to the integrity of the mitochondrial membrane. This damage promotes the release of cytochrome C into the cytoplasm and activates related apoptotic factors, collectively triggering cell apoptosis through the mitochondrial pathway (Yang L. et al., 2017). In studies focused on cell proliferation inhibition, hyperoside was shown to inhibit the activity of the Akt/mTOR/p70S6K signaling pathway in a dose-dependent manner, promoting autophagy and thereby exerting anti-tumor effects (Fu et al., 2016). Furthermore, hyperoside enhances the inhibition of lung cancer cell proliferation by reducing AMPK phosphorylation levels, upregulating the expression of caspase-3, and inducing G1 phase cell cycle arrest (Chen et al., 2020). In the realm of anti-inflammatory research, studies have demonstrated that hyperoside can significantly prevent the development of lung cancer by inhibiting the NF-κB signaling pathway, which is accompanied by a notable downregulation of inflammatory factors such as TNF-α, IL-1β, IL-6, and IL-8 (Lü, 2016). Moreover, hyperoside further amplifies the apoptotic effect by increasing the expression of pro-apoptotic proteins Bax, Bad, and Bak, while inhibiting the expression of anti-apoptotic proteins Bcl-2 and Bcl-x. Hyperoside also upregulated the expression of anti-tumor factors such as p53, p27, and p21, while inhibiting the activity of factors associated with cell proliferation and migration, including Cyclin-D1, CDK1, MMP-2, and MMP-7. This action further enhances its inhibitory effect on lung cancer cells (Liu et al., 2016). Additionally, hyperoside effectively sensitizes tumor cells to the immune system’s effects, inducing apoptosis in cancer cells through various mechanisms and inhibiting both cell proliferation and malignant transformation. However, current research is primarily confined to *in vitro* and animal studies, leaving a significant gap in our understanding of the specific effects of hyperoside on human lung cancer.

## 8 Hyperoside: actions in colon cancer

Colon cancer is among the most prevalent tumors globally. According to the latest cancer statistics, it ranks as the third most common cancer in both men and women. Furthermore, colon cancer is a significant contributor to cancer-related mortality (Siegel et al., 2024). Hyperoside has demonstrated multifaceted anticancer potential in colorectal cancer research. Both *in vivo* and *in vitro* experimental findings indicate that hyperoside can markedly inhibit tumor growth and reduce tumor mass in HCT8 xenograft mice, exhibiting greater efficacy than cisplatin (Wang et al., 2010). Additionally, hyperoside induces G2/M phase arrest in HCT8 cells when treated with varying concentrations, activates caspase-8 and caspase-3, ultimately leading to cell apoptosis (Wang et al., 2012). In in vitro studies, hyperoside also operates through dual apoptotic pathways mediated by death receptors and mitochondria in HT29 colon cancer cells. It significantly activates caspase-9 and PARP, while simultaneously upregulating the expression of the pro-apoptotic protein Bax and downregulating the anti-apoptotic protein Bcl-2 in a concentration-dependent manner, thereby triggering endogenous apoptotic signaling (Guon and Chung, 2016; Cheng et al., 2021). Hyperoside extracted from Zanthoxylum bungeanum leaves has demonstrated anti-proliferative effects in SW620 colorectal cancer cells. This compound upregulates the expression of the tumor suppressor protein p53 and the cell cycle inhibitor p21, leading to G2/M phase arrest in the cells. This process is accompanied by the accumulation of ROS, a reduction in mitochondrial membrane potential, and an upregulation of cytochrome C and Apaf-1. Additionally, hyperoside further enhances the effects of oxidative stress by significantly inhibiting the mRNA expression of glutathione peroxidase (GSH-Px) and catalase (CAT) (Zhang et al., 2017). These findings strengthen the case for hyperoside as a potential therapeutic agent against colon cancer. However, further research and systematic testing are necessary to comprehensively assess the application of hyperoside in clinical settings, particularly in clinical trials.

Conversely, the combined application of hyperoside and vincristine demonstrated significant synergy in anti-cancer treatment, effectively enhancing the anti-cancer effects. Specific manifestations of this synergy include increased activities of Caspase-3 and Caspase-9, along with a reduction in the mitochondrial membrane potential of HCT8/VCR-resistant colon cancer cells. Furthermore, this combination decreased the half inhibitory concentration (IC50) of vincristine on HCT8/VCR cells from 65.97 mg/L to 9.94 mg/L, indicating a marked improvement in the sensitivity of drug-resistant cells to the drug (Wang L. et al., 2016). In in vivo experiments, hyperoside exhibited strong anti-tumor activity in tumor-bearing mouse models, with the tumor inhibition rate in the high-dose group reaching 65.04%. This further supports its therapeutic potential in both *in vivo* and *in vitro* models (Wang et al., 2010). Additionally, high-performance liquid chromatography (HPLC) confirmed that hyperoside is the primary chemical component in lotus leaves. It can inhibit pro-survival signals in cells by down-regulating the PI3K/Akt signaling pathway, thereby reducing cell proliferation and enhancing apoptosis. This finding validates the multifaceted intervention effects of hyperoside as an effective anticancer agent (Li et al., 2022). It is clear that hyperoside is regarded as a potential therapeutic drug for colon cancer, demonstrating significant efficacy in inhibiting the survival of cancer cells. This discovery opens new research avenues for the development of novel colon cancer treatments. However, to thoroughly assess the therapeutic effects of hyperoside, additional *in vivo* experiments are necessary to establish a solid foundation for subsequent human clinical trials.

## 9 Hyperoside: actions in other cancers

**FIGURE 6:**
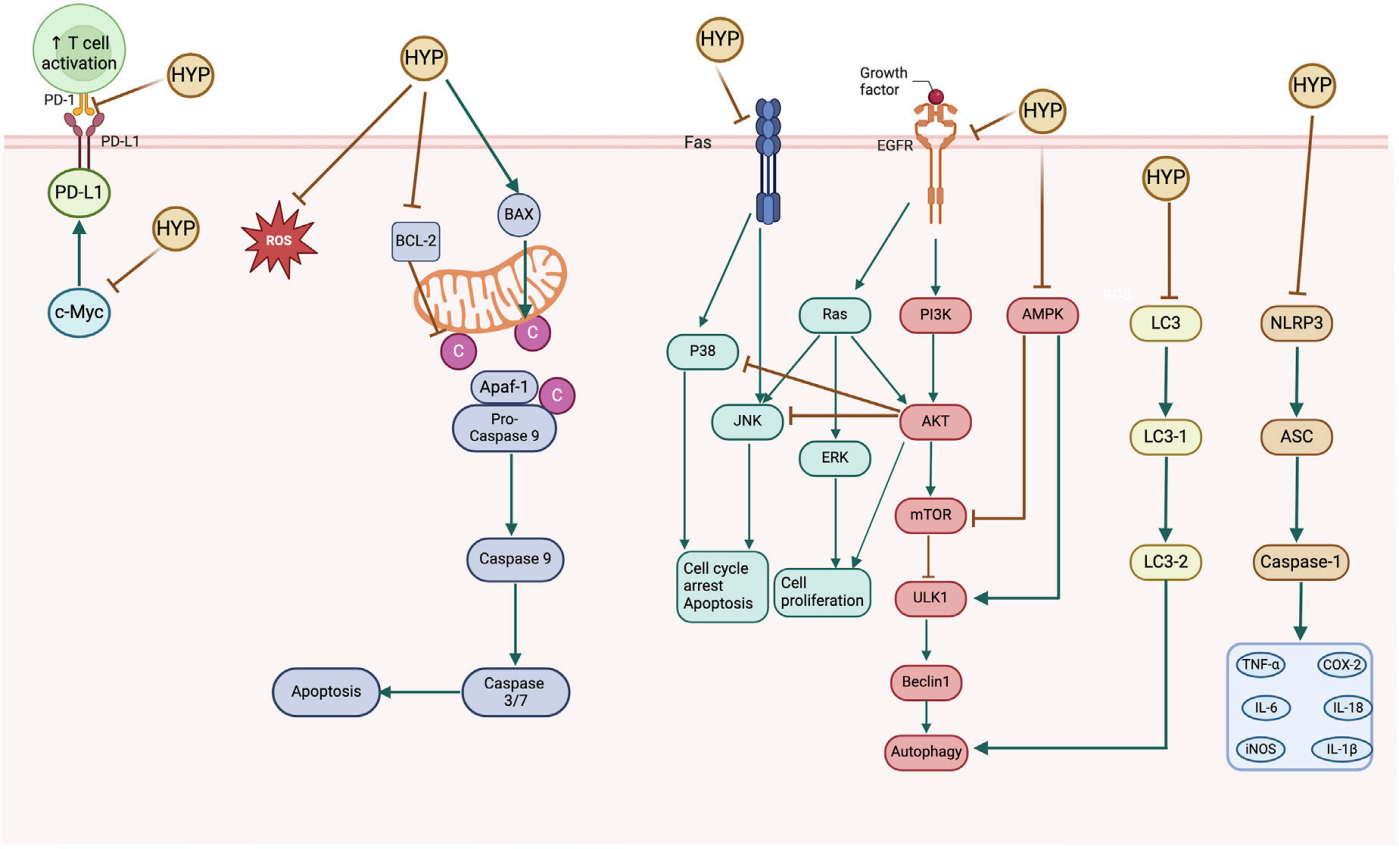
Schematic diagram of the anticancer mechanisms of hyperoside.

The primary signaling pathways implicated in the anti-cancer activity mechanism of hyperoside are illustrated in Figure 6.

### 9.1 Bladder cancer

In two bladder cancer cell lines, T24 and 5637, the application of hyperoside demonstrated a clear dose- and time-dependent effect, significantly reducing cell viability. As the treatment duration increased, the IC50 value gradually decreased. For instance, after 72 h of treatment of T24 cells, the IC50 reached 159 μM. Subsequent flow cytometry analysis indicated that hyperoside inhibited cell cycle progression by blocking the transition of cells in the G1 and G2 phases. During this process, the phosphorylation levels of key proteins, including cyclin D1 and CDK1/2, were significantly reduced. Furthermore, hyperoside was found to mildly induce cell apoptosis, activating the cleavage of caspase-3 and PARP. Through quantitative proteomics and bioinformatics analysis, it was revealed that hyperoside can activate the EGFR-Ras and Fas signaling pathways, which further regulate downstream MAPK pathways (such as ERK, JNK, and p38) and the PI3K/Akt signaling pathway, leading to cell cycle arrest and the induction of apoptosis. Additionally, animal experiments confirmed the anti-tumor effect of hyperoside in nude mouse bladder cancer transplant models (Yang et al., 2024).

### 9.2 Breast cancer

Breast cancer is the most prevalent cancer among women globally, significantly impacting the health and quality of life of many individuals (Giaquinto et al., 2024). Research addressing this issue has identified hyperoside as a promising candidate for anticancer therapy. Experimental studies, both *in vitro* and *in vivo*, have demonstrated that hyperoside effectively inhibits the viability and migration of breast cancer cells while promoting apoptosis. The mechanisms underlying this inhibitory effect are complex and multifaceted. Hyperoside disrupts NF-κB signaling by decreasing the production of ROS and down-regulating the expression of anti-apoptotic proteins such as Bcl-2 and XIAP. Additionally, it activates caspase-3 within mitochondria, leading to mitochondrial dysfunction and subsequently inducing apoptosis in breast cancer cells. Experiments conducted on subcutaneous xenograft models of breast cancer in mice revealed that hyperoside significantly inhibited tumor growth by reducing the phosphorylation levels of IκBα and p65 (Qiu et al., 2019). Importantly, as previously noted, hyperoside can enhance the sensitivity of breast cancer cells to the conventional chemotherapy drug paclitaxel, an effect mediated by the inhibition of pro-inflammatory and pro-survival mechanisms associated with TLR4 activation (Sun et al., 2020).

### 9.3 Pancreatic cancer

Pancreatic cancer is the sixth leading cause of cancer-related deaths among both genders worldwide (Bray et al., 2024). Currently, hyperoside demonstrates potential in combating pancreatic cancer through multiple mechanisms, particularly evidenced by its significant cytotoxicity against the pancreatic cancer cell lines MIA PaCa-2 and INS-1. *In vitro* studies revealed that the IC50 of hyperoside was 50 μM for MIA PaCa-2 cells and 100 μM for INS-1 cells. In MIA PaCa-2 cells, hyperoside induces cell cycle arrest in the G2/M phase, significantly activates Caspase-3, and promotes apoptosis. Additionally, the study observed that cells treated with hyperoside exhibited chromatin condensation, multinucleation, and the formation of cell membrane vesicles, alongside significant damage to the cytoskeletal structure. These observations further support the conclusion that hyperoside exerts anti-tumor effects primarily through the induction of apoptosis. The study also indicated that hyperoside increases the levels of the autophagy-related protein LC3B, suggesting a potential interaction with the autophagy mechanism. Nevertheless, the predominant mechanism of action of hyperoside appears to be the selective induction of apoptosis (Boukes and Van De Venter, 2016). Another study demonstrated that hyperoside inhibits the proliferation of BxPC-3 and PANC-1 pancreatic cancer cells. This effect may be mediated by upregulating the ratios of Bax to Bcl-2 and Bax to Bcl-xL, thereby promoting cell apoptosis. Additionally, hyperoside can inhibit the activation of the NF-κB signaling pathway and reduce the expression of related downstream genes, including survivin, c-Myc, cyclin D1, and COX-2. This mechanism has been validated in both *in vitro* experiments and *in vivo* models.

### 9.4 Liver cancer

The anti-tumor effects of hyperoside in hepatocellular carcinoma (HCC) research warrant significant attention. Numerous studies have demonstrated that hyperoside effectively inhibits the proliferation of HCC cells and induces apoptosis through various mechanisms. Its mechanisms of action include the inhibition of the PI3K/AKT signaling pathway mediated by bone morphogenetic protein 7 (BMP-7), the reduction of proteins associated with tumor cell survival (such as Cyclin D1 and c-Myc), and the regulation of the cell cycle, thereby achieving anti-proliferative effects. Furthermore, hyperoside markedly upregulates the expression of apoptosis-related proteins (such as Caspase-3 and Caspase-9) by activating the mitochondria-related P53/Caspase pathway, further promoting cell apoptosis (Jiang et al., 2018). This conclusion has been corroborated by *in vivo* experiments. In a liver cancer xenograft mouse model, the tumor volume in the hyperoside-treated group was significantly reduced, and its anti-tumor effect is closely linked to the inhibition of the PI3K/AKT signaling pathway and the regulation of apoptosis pathways (Jiang et al., 2018; Wei et al., 2021). Recent studies have also investigated the combination of hyperoside with mitochondria-targeted liposomes, which significantly enhanced the accumulation of hyperoside within the mitochondria of liver cancer cells and exhibited anti-cancer effects by activating the mitochondrial apoptosis pathway. The authors innovatively loaded hyperoside into liposomes using the thin-film hydration method, specifically employing bifunctional liposomes with pH responsiveness and mitochondrial targeting properties, such as DLD-Lip. In this method, hyperoside, phospholipids (e.g., soybean lecithin), cholesterol, and functional lipids (e.g., DSPE-PEG2000 or DSPE-Lys-DMA) are dissolved in a chloroform/methanol mixed solvent at a specific ratio. The solution is evaporated using rotary evaporation to form a uniform film, followed by vacuum drying to eliminate residual organic solvents. The dried film is then hydrated in phosphate-buffered saline (PBS) or ultrapure water, and nanoscale liposomes are prepared via sonication. Encapsulation efficiency and drug loading are assessed using HPLC to optimize formulation parameters. This drug delivery system exploits the weakly acidic tumor microenvironment, where the surface charge of liposomes transitions from negative to positive. This charge reversal enhances cellular uptake and promotes interaction with the negatively charged mitochondrial membrane, facilitating targeted drug accumulation in the mitochondria. Ultimately, this process triggers mitochondria-mediated apoptosis mechanisms, significantly improving the anti-tumor efficacy of hyperoside (Feng et al., 2021a). Notably, hyperoside also effectively protects mice from acetaminophen-induced liver damage by regulating glutathione metabolism, inhibiting the activity of the CYP2E1 enzyme, and exerting antioxidant effects (Hu et al., 2020).

### 9.5 Gastric cancer

Gastric cancer is a malignant tumor associated with high morbidity and mortality rates. Patients are at significant risk for recurrence and metastasis, as well as experiencing toxic side effects and drug resistance related to chemotherapy. These challenges substantially diminish long-term survival rates (Smyth et al., 2020). Research on various gastric cancer cell lines has demonstrated that hyperoside exerts potent inhibitory effects on these cells. For instance, in gastric cancer BGC-823 cells, hyperoside treatment resulted in an apoptosis rate of 53.15%, which was significantly higher than that of the control group and exhibited a concentration-dependent response. The mechanism underlying this inhibitory effect is closely linked to the induction of both endogenous and exogenous apoptosis, particularly through the significant upregulation of caspase-3, caspase-8, and caspase-9 activities (Sun et al., 2014). Similarly, in studies involving MKN-45 gastric cancer cells, hyperoside effectively downregulated the expression of the anti-apoptotic protein Bcl-2 and upregulated the expression of the pro-apoptotic protein Bax by inhibiting the NF-κB signaling pathway, thereby facilitating apoptosis (Lianyi, 2017). Furthermore, hyperoside inhibits the abnormal activation of the Wnt/β-Catenin signaling pathway, significantly reducing the expression levels of Wnt1 and β-Catenin while promoting the expression of tumor suppressors DKK1 and NKD1. This regulatory mechanism not only effectively suppresses the proliferation of gastric cancer cells but also significantly decreases tumor growth rates in xenograft mouse models (Ping, 2020). Hyperoside demonstrates potential anti-gastric cancer properties by regulating the cell cycle, activating apoptosis-related signaling pathways, and inhibiting NF-κB and Wnt/β-Catenin signal transduction. However, its pharmacodynamic mechanisms and prospects for clinical application *in vivo* require further validation through more extensive animal studies and clinical trials.

### 9.6 Cervical cancer

Studies have demonstrated that hyperoside exhibits toxic effects on HeLa cells. At a concentration of 100 μmol/L, the survival rate of HeLa cells decreased to 51.13%. Notable morphological changes were observed following cell treatment, including a reduction in cell size, rounded morphology, and a significant decrease in membrane gloss. Flow cytometry analysis further confirmed the pro-apoptotic effects of hyperoside (Wang et al., 2019). Hyperoside significantly downregulated the expression of the proto-oncogene c-Myc and upregulated the expression of the transferrin receptor (TFRC), thereby inhibiting the proliferation of C-33A and HeLa cells. These findings were reliably verified through bioinformatics, qRT-PCR, and Western blot experiments (Guo W. et al., 2019). Additionally, hyperoside treatment induced the upregulation of pro-apoptotic genes, such as Bax and p53, while inhibiting the expression of anti-apoptotic genes, including Bcl-2 and VEGF, and activating key apoptotic factors such as caspase-3 and caspase-8. During hyperoside treatment, the enhancement of oxidative stress resulted in decreased activities of intracellular superoxide dismutase and catalase, while levels of glutathione decreased and malondialdehyde content increased correspondingly. These changes suggest that hyperoside may influence redox status to exert its biological effects (Wang et al., 2019). The Nampt/NAD/Sirt1 pathway is closely associated with the progression of various tumors (Behrouzfar et al., 2017; Xia and Zhou, 2018; Ma et al., 2019; Bian et al., 2021). The study also found that hyperoside can effectively inhibit the activation of the nicotinamide phosphoribosyltransferase (Nampt)/nicotinamide adenine dinucleotide (NAD)/silent information regulator 1 (Sirt1) signaling pathway. This inhibition subsequently reduces the proliferation and migration of HeLa cells, a pathway that is closely associated with the progression of various tumors (Bian et al., 2021).

### 9.7 Skin cancer

The prevalence of squamous cell carcinoma (SCC) is notably higher among non-melanoma skin cancers (NMSC), with ultraviolet radiation (UVR) identified as a primary risk factor for its development (Hyeraci et al., 2023). Hyperoside may hold potential in the chemoprevention of SCC. An *in vitro* study demonstrated that hyperoside significantly inhibited the growth of various SCC cell lines, including A431, A432, and HS-4, with its effects increasing in relation to both dosage and exposure time. After 24 h of treatment, hyperoside markedly reduced the survival rate of cancer cells within a concentration range of 0–100 μM, while exhibiting no significant toxic effects on normal skin cells, thereby indicating its good selectivity. Mechanistic studies have revealed that hyperoside induces apoptosis in cancer cells by modulating the expression of key proteins. This mechanism involves the downregulation of anti-apoptotic proteins Bcl-2 and Bcl-xl, alongside the upregulation of pro-apoptotic proteins Bax and Caspase-3. Furthermore, hyperoside also promotes autophagy and enhances this process by inhibiting the PI3K/AKT/mTOR signaling pathway while activating the AMPK signaling pathway. *In vivo* experiments utilizing a 7,12-Dimethylbenz [a]anthracene/12-O-Tetradecanoylphorbol-13-acetate (DMBA/TPA)-induced skin cancer model demonstrated that hyperoside significantly reduced both the size and number of tumors. Histological analysis further indicated that hyperoside effectively reversed the epidermal thickening induced by DMBA/TPA (Jung et al., 2012; Wu and Sun, 2020).

### 9.8 Prostate cancer

The combination of hyperoside and quercetin (QH; 1:1) demonstrates potential anti-prostate cancer effects. QH significantly reduced the generation of ROS in PC3 cells and enhanced the antioxidant capacity of these cells. This effect exhibited a clear dose dependence within the concentration range of 2.5–60 μg/mL. Additionally, Western blot analysis indicated that QH effectively induces apoptosis in prostate cancer cells by activating caspase-3 and cleaving PARP. Importantly, QH significantly inhibited the invasion and migration abilities of PC3 cells, suggesting its potential role in mitigating prostate cancer metastasis. Concurrently, QH further suppresses the proliferation and survival of tumor cells by down-regulating microRNAs associated with prostate tumors, such as miR-21, while up-regulating the expression of the negatively regulated tumor suppressor programmed cell death protein 4 (PDCD4). Moreover, studies have indicated that the anti-tumor effect of QH can be partially diminished by the overexpression of miR-21, thereby reinforcing the critical role of this signaling pathway in anti-cancer mechanisms (Yang et al., 2015). Another study demonstrated that hyperoside inhibits the nuclear translocation of β-catenin by downregulating the expression of the E3 ubiquitin ligase RNF8. This process disrupts the Wnt/β-catenin signaling pathway, leading to a decrease in the expression levels of oncogenic genes associated with cancer development, such as c-myc and cyclin D1. Additionally, this mechanism significantly reduces PD-L1 levels, which may enhance the immune microenvironment and resist prostate cancer (Chen et al., 2024).

### 9.9 Osteosarcoma and multiple myeloma

Osteosarcoma (OS) is the most prevalent primary malignant bone tumor, characterized by high local invasiveness and a pronounced propensity for metastasis (Chen C. et al., 2021). Hyperoside significantly inhibited the proliferation of osteosarcoma cells (U2OS and MG63) by inducing cell cycle arrest in the G0/G1 phase. This inhibitory effect is closely associated with the increased expression levels of cell cycle regulatory factors p21 and p27, which prevent cells from transitioning from the G1 phase to the S phase. Concurrently, hyperoside markedly upregulated the expression of osteogenesis-related markers, such as Osteopontin (OPN), RUNX2, and Osteocalcin, at both the mRNA and protein levels, indicating its efficacy in inducing osteogenic differentiation of osteosarcoma cells. Immunofluorescence analysis further corroborated that Osteocalcin expression significantly increased over time. Additionally, experimental results demonstrated that hyperoside significantly activated the Smad2/3 phosphorylation signaling pathway by upregulating the expression of TGF-β and bone morphogenetic protein-2 (BMP-2). Furthermore, hyperoside notably inhibited the migration and invasion capabilities of osteosarcoma cells, suggesting that it possesses not only anti-proliferative effects but also the ability to impede the invasiveness of tumor cells. Hyperoside exhibits dual effects, acting both as an anti-proliferative agent and as a promoter of cell differentiation, without demonstrating significant cytotoxicity or apoptotic responses (Zhang et al., 2014). Additionally, another study indicated that hyperoside facilitates the mineralization and differentiation of osteoblasts, such as MC3T3-E1, while concurrently inhibiting the generation and activity of osteoclasts (Raw264.7). This suggests its impact on bone weight in in vitro experiments and highlights its regulatory role in maintaining plastic balance, which contributes to slowing down bone destruction and enhancing the bone marrow microenvironment. Specifically, hyperoside significantly downregulates the activity of the Wnt/β-catenin signaling pathway and inhibits the expression of genes associated with the proliferation and metastasis of multiple myeloma cells. Furthermore, hyperoside effectively modulates intercellular signaling and immune responses within the bone marrow microenvironment by upregulating the expression of TGF-β1, IL-6, and TNF-α. It significantly inhibits the proliferation of multiple myeloma (MM) cells, such as ARP1 and H929, by inducing a cell cycle arrest in the G0/G1 phase and reducing the levels of β-catenin protein, thereby further diminishing the proliferative activity and drug resistance of MM cells. In a mouse model of multiple myeloma, hyperoside has been shown to significantly prolong the survival time of the mice (Hou et al., 2020).

## 10 Hyperoside: actions in pulmonary fibrosis

Pulmonary fibrosis (PF) is a chronic interstitial lung disease characterized by an extremely high mortality rate. Its pathological features include the abnormal proliferation of fibroblasts, excessive deposition of extracellular matrix (ECM) in lung tissue, and persistent epithelial-to-mesenchymal transition (EMT). These pathophysiological processes result in irreversible damage to the lung tissue structure, leading to a gradual decline in gas exchange function, which may ultimately culminate in respiratory failure and death. Although the pathogenesis of PF remains incompletely understood, studies indicate that multiple factors contribute significantly to the development of fibrosis, including the TGF-β1 signaling pathway, inflammatory responses, autophagy regulation, and oxidative stress (Richeldi et al., 2017; Moss et al., 2022; Koudstaal et al., 2023). Current treatment options for idiopathic pulmonary fibrosis include pirfenidone and nintedanib. While these medications can delay disease progression to some extent, their efficacy is limited and often accompanied by significant side effects (Canestaro et al., 2016; Galli et al., 2017). Recent research has highlighted that hyperoside exhibits a range of pharmacological activities, including anti-inflammatory, antioxidant, and anti-proliferative effects, making it a subject of considerable interest in drug development. In the context of pulmonary fibrosis, hyperoside has shown significant anti-fibrotic potential through a multi-target mechanism.

### 10.1 EMT

EMT is a critical process in the development and progression of pulmonary fibrosis. EMT involves the transformation of epithelial cells into mesenchymal cells, a process significantly influenced by profibrotic factors such as TGF-β. During this transformation, epithelial cells lose their polarity and enhance their migratory and invasive capabilities, thereby accelerating the pathological changes associated with pulmonary fibrosis (Meng et al., 2016; Hu et al., 2018; Griggs and Lemmon, 2023; Sun et al., 2023). Consequently, inhibiting the progression of EMT is regarded as a vital strategy for mitigating the onset of fibrosis. Research indicates that hyperoside can markedly inhibit TGF-β1-induced EMT, positively impacting the development of pulmonary fibrosis. In experiments conducted with A549 cells, hyperoside and its derivatives (such as Z6) effectively blocked EMT induced by TGF-β1 by upregulating the expression of the epithelial cell marker E-cadherin and downregulating the mesenchymal cell marker vimentin. E-cadherin, a key protein in intercellular adhesion, plays a crucial role in maintaining cell polarity and integrity, while the expression of vimentin signifies the initiation of EMT. Furthermore, experimental data analyzed through RT-qPCR and Western blotting corroborate these findings, indicating that hyperoside can effectively inhibit the morphological changes and migratory capabilities of alveolar epithelial cells, thereby preventing the progression of fibrosis (Gao et al., 2024).

### 10.2 Inflammation and oxidative stress

Oxidative stress and the inflammatory response are significant pathological mechanisms contributing to pulmonary fibrosis. During the pathological progression of pulmonary fibrosis, cell damage induced by oxidative stress and the excessive release of inflammatory factors not only directly compromise the structural integrity of lung tissue but also further exacerbate the development of fibrosis (Cameli et al., 2020). Consequently, mitigating oxidative stress and the inflammatory response is anticipated to effectively manage the progression of fibrosis. The bleomycin-induced mouse model of pulmonary fibrosis is widely employed to assess the anti-fibrotic effects of various pharmacological agents (Jenkins et al., 2017; Kolb et al., 2020). One study found that hyperoside exhibited significant antioxidant and anti-inflammatory effects, effectively alleviating the pathological process of pulmonary fibrosis. Specifically, hyperoside decreased the levels of the oxidative stress marker malondialdehyde (MDA) and reduced the expression of pro-inflammatory cytokines TNF-α and IL-6 in lung tissue, which are associated with inflammatory responses and fibrosis. This deterioration is closely related to the effects of globalization. SOD is a key antioxidant enzyme whose primary function is to eliminate excess superoxide anion free radicals in the body, thereby mitigating oxidative damage to cells (Wang Y. et al., 2018). Hyperoside significantly enhanced the activity of SOD, effectively reducing bleomycin-induced lung tissue damage. Additionally, the study found that hyperoside significantly inhibited the EMT process induced by TGF-β1 by blocking the AKT/Glycogen Synthase Kinase 3 β(GSK3β) signaling pathway, further preventing the progression of fibrosis. Histological analysis corroborated the anti-fibrotic effect of hyperoside. In HE-stained and Masson’s trichrome-stained tissue sections, mice treated with bleomycin exhibited marked alveolar structural damage and excessive collagen deposition. Following hyperoside treatment, these fibrotic features were significantly alleviated, leading to notable improvements in the health of lung tissue (Huang et al., 2020).

### 10.3 Upregulation of autophagy levels

Autophagy regulation plays a crucial role in the pathogenesis of pulmonary fibrosis (Racanelli et al., 2018; Ornatowski et al., 2020). However, the use of autophagy inducers alone to treat pulmonary fibrosis may compromise the stability of epithelial cells, potentially exacerbating lung damage (Mikaelian et al., 2013; Peng et al., 2022). To address this issue, researchers have innovatively developed a combined delivery system designed to co-load hyperoside and rapamycin into macrophages, thereby achieving a synergistic therapeutic effect on pulmonary fibrosis (Wang et al., 2024).

**FIGURE 7:**
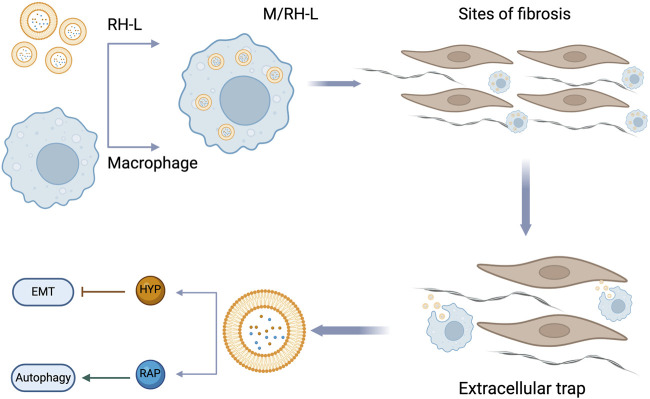
Delivery mechanism of macrophage-loaded hyperoside and rapamycin.

This macrophage delivery system is characterized by unique design features and mechanisms of action. Specifically, hyperoside and rapamycin were successfully encapsulated within macrophages, which are highly chemotactic immune cells that respond to chemical signals released from fibrotic areas, allowing them to migrate precisely toward the lesion (Weng et al., 2022). By leveraging this property, hyperoside and rapamycin are effectively delivered to fibrotic regions. Within these areas, macrophages release the encapsulated drugs in the form of liposomes by forming extracellular traps. This release mechanism not only enhances the local concentration of the drugs within the fibrotic area but also improves the sustained maintenance of the drugs at the target site, thereby reducing potential adverse effects on healthy tissue (Ma et al., 2016; Jensen et al., 2023). Compared to traditional drug delivery methods, the macrophage delivery system enhances drug targeting and improves the stability and durability of treatment through localized release. In this system, rapamycin functions as an autophagy inducer, targeting the mTOR signaling pathway to activate the cellular autophagy process. Autophagy is a critical mechanism for the degradation of damaged proteins and cellular waste, playing an essential role in restoring cellular homeostasis. Concurrently, hyperoside mitigates excessive fibroblast proliferation and abnormal collagen deposition by inhibiting the EMT process. Inhibiting EMT is vital for preventing the further progression of fibrosis. Thus, the combined application of these two drugs produces a synergistic effect: rapamycin enhances cellular clearance by activating autophagy, while hyperoside protects tissue structure from further damage by inhibiting EMT, collectively slowing the pathological progression of pulmonary fibrosis. Subsequent experiments revealed that the alveolar structure was repaired, and collagen deposition was significantly reduced. Additionally, fibroblast proliferation was effectively inhibited. These findings indicate that the combined delivery system of hyperoside and rapamycin co-loaded into macrophages (M/RH-L) presents an innovative and effective drug delivery strategy for improving pulmonary fibrosis (Wang et al., 2024) (Figure 7).

### 10.4 Others

The potential application of hyperoside in various fibrotic diseases has garnered increasing attention.

Relevant studies have demonstrated that hyperoside can significantly enhance cardiac function in a rat model of acute heart failure (ACF). Specifically, hyperoside is shown to increase left ventricular ejection fraction (LVEF), reduce left ventricular end-diastolic pressure (LVEDP), and decrease the ratio of heart weight to body weight. These findings suggest that hyperoside may mitigate heart failure by improving cardiac function. Furthermore, this compound exhibits promising protective effects on the liver, significantly reducing the area of liver fibrosis and hydroxyproline content, while alleviating edema and vacuolar degeneration of liver cells. Mechanistic studies have revealed that hyperoside can diminish the expression of fibrosis-related factors by inhibiting the TGF-β1/Smad signaling pathway, thereby preventing the activation of hepatic stellate cells and delaying the progression of liver fibrosis (Guo X. et al., 2019). In a carbon tetrachloride (CCl4)-induced liver fibrosis model, hyperoside alleviates liver fibrosis symptoms by modulating the poly (ADP-ribose) polymerase 1 (PARP-1) and high-mobility group box 1 protein (HMGB1) signaling pathways, counteracting damage caused by oxidative stress and inflammation, and thereby further enhancing its anti-fibrotic effects (Zeng et al., 2023). The study demonstrated that CCl4 treatment resulted in a decrease in the expression level of the liver antioxidant transcription factor Nrf2. In contrast, hyperoside enhanced its biological activity by promoting the nuclear translocation of Nrf2 and activating SOD and GSH-Px, thereby providing a stronger antioxidant defense for the liver. Furthermore, MDA levels were significantly reduced following hyperoside treatment, further underscoring its potential to mitigate lipid peroxidative damage (Zou et al., 2017).

Hyperoside has demonstrated significant potential in the study of renal fibrosis, particularly in the unilateral ureteral obstruction (UUO) model (Yan et al., 2014). Relevant studies indicate that the combination of hyperoside and quercetin (QH) can markedly inhibit the expression of renal fibrosis-related markers, such as α-Smooth Muscle Actin (α-SMA) and Fibronectin (FN). Further experiments have confirmed that the QH combination effectively reduces the expression of these markers, thereby reflecting its inhibitory effect on renal interstitial fibrosis. Additionally, *in vitro* experiments have further validated the efficacy of the QH combination. In a mesangial cell model induced by white IL-1β, QH exhibited an inhibitory effect on the expression of FN and α-SMA. The progression of renal fibrosis is closely associated with the deposition of extracellular matrix, and the QH combination may mitigate this progression through multiple mechanisms, including the inhibition of pro-inflammatory signals and the reduction of oxidative stress. Furthermore, hyperoside also plays a beneficial role in reducing endometrial fibrosis. Its intervention can enhance the intimal microenvironment and inhibit the excessive fibrotic response by suppressing the activation of the TGF-β/Smad3 signaling pathway (Zhu Z. et al., 2022).

## 11 Hyperoside: actions in diabetes mellitus

Diabetes is not only classified as a metabolic disease but also recognized as a complex systemic condition. Although its primary characteristic is a disorder in blood sugar regulation, research has demonstrated that diabetes impacts multiple systems, including the cardiovascular, nervous, and immune systems, resulting in damage to various organs throughout the body. In recent years, the global incidence of diabetes has continued to rise, affecting over 400 million individuals. Concurrently, diabetes-related complications have significantly diminished patients’ quality of life and longevity (Cole and Florez, 2020; Ceriello and Prattichizzo, 2021; Demir et al., 2021). Traditional research has primarily focused on insulin functional defects; however, emerging perspectives suggest that the development of diabetes arises from a multi-system network imbalance, particularly evident in the interplay between chronic inflammation, oxidative stress, and metabolic network abnormalities (An et al., 2023; Yi et al., 2024). While obesity is regarded as a major risk factor for diabetes, the relationship between the two is not strictly linear. Although relevant studies have extensively investigated the dysregulation of adipose tissue and insulin resistance in obese patients, a considerable number of obese individuals do not develop diabetes, and some non-obese patients exhibit diabetic characteristics (Malone and Hansen, 2019; Pérez-Torres et al., 2021; Aggarwal et al., 2023). Research indicates that abnormalities in lipid metabolism, such as diacylglycerols and sphingolipids, may play a central role in the pathogenesis of diabetes. These metabolites directly disrupt the insulin signaling pathway and are independently associated with body weight (Athyros et al., 2018; Xu et al., 2018; Petrenko et al., 2023). The traditional model of diabetes management primarily emphasizes glucose-lowering objectives; however, this approach overlooks the systemic characteristics inherent to diabetes. Contemporary treatment paradigms advocate for a more holistic management strategy that prioritizes the regulation of oxidative stress, chronic inflammation, and the prevention of multi-system complications. Innovative exercise modalities, including high-intensity interval training (HIIT) and resistance training, have been shown to enhance blood glucose levels by promoting GLUT4 transport while simultaneously improving patients’ quality of life (Chavanelle et al., 2017; Sjöros et al., 2018; Kartinah et al., 2024). Furthermore, personalized exercise and dietary plans informed by artificial intelligence technology have demonstrated significant improvements in patient compliance (Ellahham, 2020). In the realm of pharmacotherapy, novel SGLT2 inhibitors and GLP-1 agonists not only effectively lower blood glucose levels but also offer the dual advantages of cardiovascular protection and weight management. Currently, natural products such as hyperoside are emerging as promising alternatives to high-cost medications due to their multi-target activity and notable anti-inflammatory and antioxidant properties. Oxidative stress and inflammation are critical pathological mechanisms involved in the onset and progression of diabetes (Ma et al., 2018). There exists a close interplay between these two factors at both molecular and systemic levels. In a hyperglycemic state, cells generate excess ROS, which can lead to mitochondrial dysfunction and cellular damage. Concurrently, the elevation of reactive oxygen species activates pro-inflammatory pathways, such as NF-κB, thereby instigating a chronic low-grade inflammatory response. The inflammatory response exacerbates insulin resistance and the functional decline of pancreatic β-cells by secreting pro-inflammatory cytokines, such as TNF-α and IL-6, thereby creating a vicious cycle (Volpe et al., 2018; Oguntibeju, 2019; Tsalamandris et al., 2019). Researchers have identified hyperoside from Myrica rubra (MR) using HPLC technology, recognizing it as one of the key active constituents of the plant. Additionally, α-glucosidase inhibition experiments demonstrated that hyperoside exhibits a significant hypoglycemic effect (Chang et al., 2022). One study reported that hyperoside reduced blood glucose levels in a dose-dependent manner in a rat model of streptozotocin-induced diabetes, underscoring its potential clinical application. Nevertheless, researchers assert that further clinical trials are essential to validate its efficacy (Verma et al., 2013).

Neuropathy, retinopathy, and diabetic nephropathy (DN) are the most prevalent microvascular complications affecting patients with diabetes (Mauricio et al., 2020). Research indicates that hyperoside may indirectly influence the overall progression of diabetes by modulating these diabetes-related complications. Hyperoside exhibits superior anti-inflammatory and antioxidant properties, effectively improving oxidative stress and inflammatory damage in diabetic rats (DG rats) (Wu W. et al., 2020). The combination of a high-fat diet and streptozotocin (STZ) injection is widely recognized as a classic model for inducing diabetic retinopathy (Oyake et al., 1996; Maines et al., 2006). Studies conducted using this model demonstrated that diabetic rats treated with hyperoside exhibited significantly lower fasting blood glucose (FBG) levels compared to the model group, while retinal damage was alleviated and the number of cells surrounding capillaries increased (Wu W. et al., 2020). Furthermore, researchers discovered that hyperoside significantly mitigates the apoptosis of rat retinal vascular endothelial cells (RVECs) induced by oxidative damage (Wu et al., 2013). Apoptosis is regarded as one of the primary causes of nerve cell death in the early stages of diabetic retinopathy (Li et al., 2023). The findings suggest that hyperoside’s effect on cell proliferation is not achieved through direct stimulation of the cells, but rather by reducing the damage to the vitality of RVECs in a high-glucose environment. Additionally, experimental results indicated that hyperoside could significantly decrease the apoptosis rate of RVECs in DG rats in a dose-dependent manner (Wu W. et al., 2020). Through qPCR and Western blot experiments, this study further elucidated the significant role of hyperoside in regulating molecules associated with the apoptosis signaling pathway, including Bax, Bcl-2, and CytoC. Notably, compared to the untreated DG group, RVECs in the hyperoside-treated DG group exhibited a higher cell survival rate and an increased proportion of cells in the S-phase, with this effect being distinctly dose-dependent. In a high-glucose environment, the activation of the TGF-β1/miR-200b/VEGF signaling pathway is critical for the excessive proliferation and angiogenesis of retinal endothelial cells (Jiang et al., 2015; Li et al., 2017; Pang et al., 2020). The study demonstrated that hyperoside could significantly reduce both the mRNA and protein expression levels of TGF-β1 and VEGF, while also promoting the restoration of miR-200b expression, irrespective of concentration. Furthermore, hyperoside treatment resulted in a reduction in the number of retinal capillaries and a decreased endothelial cell to pericyte ratio (E/P ratio). Histological analysis through HE staining and retinal tissue section examination indicated that hyperoside can effectively ameliorate the pathological changes observed in the retinas of diabetic rats, as well as alleviate the proliferation, migration, and angiogenesis of retinal endothelial cells (Yu et al., 2023). Given its impressive efficacy, hyperoside emerges as a promising candidate for the treatment of diabetic retinopathy.

Currently, numerous studies investigate the effects of hyperoside on diabetic nephropathy (DN). Hyperoside demonstrates significant renal protective effects in STZ-induced diabetic nephropathy models, with its mechanisms involving multiple cellular and molecular pathways. Firstly, hyperoside can decrease urinary albumin excretion levels, an important feature of early DN. This effect primarily occurs through the inhibition of the overexpression of caspase-3 and caspase-8 induced by advanced glycation end products (AGEs), thereby reducing podocyte apoptosis and maintaining the integrity of the glomerular filtration barrier (Zhou et al., 2012). Furthermore, it is well-established that miRNAs are extensively utilized in the clinical diagnosis and treatment of diabetic nephropathy. As key biomarkers, the regulation of their expression levels significantly influences the onset and prognosis of diabetic nephropathy (He et al., 2021). Hyperoside effectively inhibits high glucose-induced podocyte damage by modulating the miR-499-5p/APC axis, which leads to a reduction in the accumulation of extracellular matrix components, such as type I and type IV collagen. Additionally, it inhibits the expression of pro-inflammatory factors, including TNF-α, MCP-1, and IL-1β, as well as cell apoptosis, potentially mitigating kidney damage through the activation of the Wnt/β-catenin signaling pathway (Zhou et al., 2021a). In a diabetic mouse model, hyperoside significantly improved glomerular hypertrophy, matrix expansion, and collagen deposition, while also enhancing renal function parameters, thereby suggesting its therapeutic potential for diabetic nephropathy. Furthermore, hyperoside effectively alleviates high glucose-induced apoptosis and inflammatory responses in renal tubular epithelial cells (HK-2) by regulating the miR-499a-5p/NRIP1 signaling pathway (Zhou et al., 2021b). Hyperoside also significantly inhibited the proliferation of glomerular mesangial cells (SV40-MES13) induced by high glucose in a dose-dependent manner. Its mechanism of action primarily involves the downregulation of the ERK/CREB/miR-34a signaling pathway, which reduces ERK phosphorylation and miR-34a expression. This mechanism inhibits mesangial cell proliferation without inducing cell death (Zhang et al., 2020). Further studies have demonstrated that hyperoside restores the integrity of the glomerular basement membrane (GBM) by inhibiting the promoter activity of the heparinase gene (HPR1) and reducing both the mRNA and protein levels of heparanase. Hyperoside effectively reverses the degradation of heparan sulfate (HS) induced by high glucose levels and ROS, as well as the thickening of the GBM, thereby alleviating disorders in filtration barrier function and reducing proteinuria (An et al., 2017). It is important to note that the direct regulatory mechanism of hyperoside on the HPR1 promoter requires further investigation to clarify its specific role. Immune regulation is a significant mechanism through which hyperoside exerts its effects in diabetic nephropathy (DN). The activation of macrophages is critical in renal inflammatory damage associated with DN, as these cells are among the most abundant innate immune cells present in the renal tissue of DN patients (Anders and Ryu, 2011; Yang and Mou, 2018). Hyperoside significantly inhibits inflammatory signal activation and decreases the levels of inflammatory factors, including MCP-1, TNF-α, and inducible nitric oxide synthase (iNOS), by promoting the polarization of macrophages from the pro-inflammatory M1 phenotype to the anti-inflammatory M2 phenotype. This shift is accompanied by an increase in the expression of anti-inflammatory factors such as Arg-1, CD163, and CD206, ultimately alleviating glomerular matrix expansion and proteinuria. Hyperoside also promotes the differentiation of Th2 type (CD4^+^IL-4^+^) and Treg type (CD4^+^Foxp3^+^) cells by regulating the balance between T cell subsets, thereby modulating the Th1/Th17 and Th2/Treg immune responses. This regulation further mitigates inflammatory damage to the kidneys (Liu et al., 2021). Research indicates that the progression of diabetic nephropathy (DN) is closely associated with the morphological and functional alterations of podocytes, with mitochondrial dysfunction and abnormal division identified as key mechanisms contributing to podocyte damage (Guan et al., 2015; Audzeyenka et al., 2022). Regarding mitochondrial protection, hyperoside significantly alleviates doxorubicin (ADR)-induced albuminuria and podocyte injury. It inhibits mitochondrial fission and maintains mitochondrial dynamics by regulating the dephosphorylation and mitochondrial translocation of the key protein Drp1, which is involved in mitochondrial fission. Additionally, hyperoside restores mitochondrial membrane potential, reduces the generation of ROS, and enhances the replication capacity of mitochondrial DNA. These mechanisms collectively contribute to the protection of podocyte function (Chen et al., 2017). Hyperoside effectively mitigates damage associated with oxidative stress and pyroptosis in DN by modulating the interaction between ROS and the ERK1/2 signaling pathway. Its mechanism of action is primarily evident in two aspects: first, hyperoside reduces the production of ROS, thereby alleviating intracellular oxidative stress; second, it inhibits the hyperphosphorylation of ERK1/2, which significantly decreases the expression of the pyroptosis marker GSDMD-NT (Zhang et al., 2023). These combined effects lead to improvements in tubulointerstitial fibrosis and epithelial cell damage. Furthermore, results from animal studies have corroborated that hyperoside demonstrates significant efficacy in the prevention and treatment of diabetic nephropathy through multiple mechanisms, including antioxidant, anti-inflammatory, and anti-fibrotic actions (Yi-xuan et al., 2024).

Fatty acid binding protein 4 (FABP4), an adipokine, plays a crucial role in regulating lipid transport and maintaining metabolic balance in mature adipocytes. The overexpression of Fatty Acid-Binding Protein 4(FABP4) is closely associated with the development of various metabolic diseases, including diabetes and insulin resistance (Chung et al., 2019; Dahlström et al., 2021). Research indicates that inhibiting FABP4 activity can effectively reduce body weight and enhance insulin sensitivity in obesity models, thereby reinforcing the potential of FABP4 as a target for intervention in metabolic diseases (Chung et al., 2021). Molecular docking experiments have identified hyperoside as an inhibitor with a high binding affinity for FABP4, demonstrating significant inhibition of FABP4 activity, which positions it as a promising candidate for the development of anti-diabetic drugs. Functional studies reveal that hyperoside promotes lipid accumulation and upregulates the expression of peroxisome proliferator-activated receptor γ (PPARγ) protein during the differentiation of mouse 3T3-L1 preadipocytes. Further experimental results indicate that the application of PPARγ antagonists or FABP4 overexpression markedly attenuates hyperoside-induced adipogenesis, suggesting that its mechanism of action may operate through the FABP4/PPARγ signaling pathway (Wang Y. et al., 2016). Based on this analysis, we strongly believe that hyperoside exerts a beneficial effect on diabetes and its related complications. However, it is imperative to conduct additional clinical trials focusing on hyperoside within the realm of diabetes research.

## 12 Hyperoside: actions in PD and AD

### 12.1 Hyperoside: actions in PD

Parkinson’s Disease (PD) is a chronic neurological disorder characterized by degenerative changes in the central nervous system. The primary pathological mechanism of the disease involves the selective loss of dopaminergic neurons in the substantia nigra of the midbrain, leading to a range of typical motor symptoms, including tremor, muscle rigidity, bradykinesia, and postural instability (Kalia and Lang, 2015). Currently, researchers commonly utilize compounds such as 1-methyl-4-phenyl-1,2,3,6-tetrahydropyridine (MPTP) and 6-hydroxydopamine (OHDA) to induce neurotoxicity and oxidative stress, thereby simulating the neuropathological manifestations of Parkinson’s disease (Miyazaki et al., 2020; Siracusa et al., 2020; Innos and Hickey, 2021).

Hyperoside demonstrated significant neuroprotective effects in the 6-Hydroxydopamine(6-OHDA)-induced human SH-SY5Y dopaminergic neuron injury model (Kwon et al., 2019). Specifically, this compound effectively mitigated the decrease in cell viability, the increase in lactate dehydrogenase (LDH) release, the excessive accumulation of ROS, and the disruption of mitochondrial membrane potential caused by 6-OHDA. Furthermore, the study systematically revealed for the first time that hyperoside alleviates 6-OHDA-induced neurotoxicity by activating the Nrf2-HO-1 signaling pathway, which plays a crucial role in reducing neuronal death due to oxidative stress (Kwon et al., 2019). Additional research further supports the neuroprotective effects of hyperoside. The findings indicated that hyperoside positively influenced the Parkinson’s disease rat model and SH-SY5Y cells induced by rotenone by modulating the oxidative stress response and the balance between autophagy and apoptosis (Fan et al., 2021). Rotenone, a well-known inhibitor of mitochondrial complex I, selectively targets the substantia nigra pars compacta (SNpc) of the striatum and the respiratory chain, potentially damaging mitochondrial function and leading to increased cellular oxidative stress, thereby initiating a mitochondrial-mediated apoptosis pathway (Johnson and Bobrovskaya, 2015). Hyperoside intervention significantly increased the number of dopamine neurons in the substantia nigra of rats, improved their behavioral deficits, inhibited the expression of apoptosis-related proteins Bax, caspase 3, and cytochrome C, and promoted the expression of the anti-apoptotic protein Bcl-2. Additionally, it was observed that hyperoside reduced the expression of autophagy-related proteins Beclin1, LC3II, and p62. Notably, the viability of SH-SY5Y cells was not significantly affected when treated with hyperoside at various concentrations (0, 0.25, 0.5, 1, and 2 μM). Furthermore, the study found that the application of the autophagy agonist rapamycin could reverse the protective effect of hyperoside on rotenone-induced cell damage. This finding further substantiates the hypothesis that hyperoside protects neurons through two pathways: autophagy and apoptosis (Fan et al., 2021). The occurrence and progression of Parkinson’s disease are closely linked to neuroinflammation, wherein excessive activation of microglia is considered a significant driving factor of inflammation-mediated neurotoxicity. This activation triggers the release of a series of pro-inflammatory cytokines and mediators, such as IL-1β, TNF-α, and NO (Taka et al., 2015; Marogianni et al., 2020). Recent studies have demonstrated that in experiments involving LPS-induced microglia, hyperoside significantly reduces the secretion of IL-1β and TNF-α, while downregulating the transcription of pro-inflammatory genes and inhibiting the expression of iNOS, thereby decreasing the production of the neurotoxic substance NO. Additionally, results from western blotting experiments and electrophoretic mobility shift assays (EMSA) indicated that hyperoside inhibits the excessive activation of microglia primarily by targeting the p38 MAPK and NF-κB signaling pathways (Fan et al., 2017).

Interestingly, the research team discovered that the administration of 20 μM hyperoside did not exhibit significant protective effects against neuronal toxicity directly induced by MPP+ (the active neurotoxic metabolite of MPTP) (Fan et al., 2017). This observation may be associated with the concentration of hyperoside. Furthermore, results from another research team indicated that hyperoside (100 μg/mL) can mitigate the cytotoxicity of SH-SY5Y cells caused by MPTP/MPP+ *in vitro* (Xu et al., 2023). Additionally, in both the MPTP-induced C57BL/6 mouse model and the MPP+-induced SH-SY5Y cell model, hyperoside treatment resulted in a reduction of reactive oxygen species ROS, NO, H_2_O_2_, and MDA levels, both *in vivo* and *in vitro*. Moreover, hyperoside not only alleviates oxidative stress and mitochondrial damage but also significantly enhances the levels of BDNF, glial cell-derived neurotrophic factor (GDNF), and cerebral dopamine neurotrophic factor (CDNF), thereby further augmenting the protective effect on dopaminergic neurons. This conclusion was verified using the ELISA method. Observations from the DigiGait gait analysis system and histological analysis indicated that MPTP-induced Parkinson’s disease mice exhibited improvements in behavioral impairments within 14 days following hyperoside injection. Furthermore, there was a significant restoration in the number of Tyrosine Hydroxylase (TH)+ cells. The formation of TH-catalyzed dopamine (DA) precursors is directly linked to the synthesis and secretion of DA; thus, the recovery of TH+ cell numbers suggests a reduction in the extent of dopaminergic neuron loss (Xu et al., 2023). Recent studies have identified the NOD-like receptor family pyrin domain containing 3(NLRP3) inflammasome as a critical driver of microglial activation and neuroinflammation associated with Parkinson’s disease. Its abnormal activation can lead to the release of pro-inflammatory factors, such as IL-1β and IL-18, which may accelerate the degeneration of dopaminergic neurons (Anderson et al., 2021). The researchers employed an MPTP-induced Parkinson’s disease mouse model and conducted a series of behavioral experiments, including the rotarod and pole tests, to assess the efficacy of hyperoside. The experimental results demonstrated that hyperoside significantly alleviated motor dysfunction in the mouse models. Histological analysis further revealed that hyperoside markedly reduced the loss of dopaminergic neurons in the substantia nigra pars compacta (SNpc) and inhibited the overactivation of astrocytes and microglia, as confirmed by Glial Fibrillary Acidic Protein (GFAP) and Ionized Calcium-Binding Adaptor Molecule 1(IBA-1) immunohistochemical staining. Concurrently, *in vitro* experiments investigated the specific mechanism of action of hyperoside, revealing that it inhibits the activation of the NLRP3 inflammasome through the activation of the pituitary adenylate cyclase-activating peptide (PACAP)-cAMP Response Element-Binding Protein (CREB) pathway, thereby exerting an anti-inflammatory effect (Wang et al., 2022). A recent study on Parkinson’s disease has reaffirmed that hyperoside exhibits dual mechanisms of neuroprotection and anti-inflammation, effectively mitigating the pathological damage associated with the disease (Xu X. et al., 2022). Furthermore, another investigation validated its efficacy through both *in vivo* and *in vitro* approaches. In a model of PD mice, treatment with hyperoside significantly restored the expression levels of TH, a marker associated with dopaminergic neurons, while simultaneously decreasing the expression of iNOS and increasing the proportion of anti-inflammatory markers, such as Arg-1. Additionally, hyperoside reduced the levels of inflammatory factors, including TNF-α and IL-1β, and inhibited the inflammatory cascade by activating the AKT signaling pathway. In a LPS-induced microglial BV2 model, hyperoside also regulated the M1/M2 polarization state of microglia, effectively inhibiting the inflammatory response and reducing neuronal damage (Fan et al., 2024).

In summary, hyperoside mitigates neuroinflammation and nerve damage associated with Parkinson’s disease by regulating the balance between autophagy and apoptosis, while also exerting anti-inflammatory and antioxidative stress effects. It is important to note that, although there are several related studies in the existing literature, further research is necessary to thoroughly and comprehensively evaluate the efficacy of hyperoside and to facilitate its promotion as a viable treatment option for Parkinson’s disease in broader clinical applications. A more in-depth discussion and additional clinical experimental data are warranted.

### 12.2 Hyperoside: actions in AD

As the global population gradually ages, the incidence of Alzheimer’s disease (AD) has risen significantly. Relevant predictions indicate that by 2050, the number of individuals affected by this disease may reach 152 million (Scheltens et al., 2021). AD is a prevalent neurodegenerative disorder and one of the leading causes of dementia. The hallmark of the disease is the progressive decline in cognitive function, which manifests as impairments in memory, language skills, thinking abilities, and executive function. Furthermore, the accumulation of β-amyloid (Aβ) and abnormal phosphorylation of tau protein have been identified as critical pathological features of AD. Concurrently, oxidative stress, mitochondrial dysfunction, and damage to the blood-brain barrier are also believed to play significant roles in the onset and progression of Alzheimer’s disease. Currently, the diagnosis of AD primarily relies on biomarkers and imaging examinations, while treatment strategies mainly aim to alleviate symptoms, with no effective cure identified to date (Twarowski and Herbet, 2023).

Hyperoside has demonstrated significant neuroprotective effects in cell experiments related to AD, with potential mechanisms involving multiple cellular biological effects, including antioxidant and anti-inflammatory properties. This provides a crucial scientific basis for its prospective application in the treatment of AD. In the study of neurodegenerative diseases such as AD and PD, PC12 cells are widely regarded as a classic model for investigating reactive oxygen species-related pathologies. Chemical reagents such as hydrogen peroxide (H_2_O_2_) and tert-butyl hydroperoxide (t-BuOOH) are frequently employed to induce oxidative stress and apoptosis (Cesquini et al., 2003; Twarowski and Herbet, 2023). Experimental results obtained through these methods indicate that hyperoside effectively alleviates cell apoptosis and helps prevent morphological changes, such as cell shrinkage. Furthermore, experiments have revealed that hyperoside significantly enhances cell survival rates in a dose-dependent manner. Hyperoside has also been shown to inhibit the release of LDH, a marker of cell membrane damage, thereby maintaining cell membrane integrity. Its mechanism may be associated with the scavenging of free radicals and the chelation of transition metal ions, which further inhibits the generation of free radicals and the diffusion of chain reactions (Liu et al., 2005). Additionally, using cytotoxicity assays induced by Aβ (25–35) and Aβ (1–40), research findings demonstrated that pretreatment with hyperoside significantly improved the survival rate of microglia exposed to Aβ, while reducing oxidative stress-induced apoptosis and damage caused by excessive reactive oxygen species production triggered by Aβ. Aβ disrupts the structure and function of cell membranes by interfering with their fluidity. Hyperoside can partially reverse this membrane damage, effectively maintaining the integrity and function of cell membranes while reducing the risk of AD (Kraus et al., 2007). Another study utilized Aβ(1–42) to induce apoptosis and blood-brain barrier damage in mouse brain microvascular endothelial cells (bEnd.3). Hyperoside operates through multiple protective mechanisms, including the inhibition of mitochondria-related cell death, endoplasmic reticulum stress, and death receptor pathways. Additionally, it protects tight junction proteins (TJs) and inhibits the activity of matrix metalloproteinases (MMPs), thereby safeguarding brain microvascular endothelial cells and ensuring the integrity of the blood-brain barrier is preserved (Liu et al., 2018). Hyperoside significantly ameliorates the neurotoxicity induced by Aβ(25–35) in primary cultured rat cortical neurons by modulating the PI3K/Akt/Bad/BclXL signaling axis. This protective effect is evidenced by a reduction in typical apoptotic characteristics, such as nuclear condensation, formation of apoptotic bodies, and disruption of membrane integrity. Furthermore, hyperoside effectively repairs the loss of mitochondrial membrane potential and reduces excessive ROS production in mitochondria, thereby maintaining mitochondrial membrane integrity (Zeng et al., 2011). As a neurotransmitter, glutamate’s neurotoxicity primarily arises from its ability to elevate levels of reactive oxygen species and reactive nitrogen species (RNS) in nerve cells. Hyperoside has been shown to significantly mitigate glutamate-induced ROS generation, inhibit mitochondrial dysfunction associated with oxidative damage, and alleviate neuronal injury resulting from the overexpression of inflammatory factors (Xin et al., 2019). Additionally, researchers discovered that hyperoside can reduce LPS-induced apoptosis in HT22 cells by modulating the expression of SIRT1 and activating the Wnt/β-catenin and Sonic Hedgehog signaling pathways, thereby addressing inflammatory responses and oxidative stress damage while promoting the recovery of neurotrophic factors (Huang J. et al., 2021). Notably, another study indicated that hyperoside can enhance the expression of the SIRT1 protein, thereby providing protection to ECV-304 cells against t-BuOOH-induced damage (Li et al., 2008).

An in-depth study of the application of hyperoside in animal models can reveal its potential mechanisms of action and facilitate its translation into clinical applications. Scopolamine is known to induce memory impairment in mice (Xie et al., 2023). In related animal experiments, oral administration of hyperoside at a dose of 2.5 mg/kg for seven consecutive days significantly improved memory impairment induced by scopolamine. In the passive avoidance experiment, the latency of mice to enter the dark room was significantly prolonged, with effects comparable to those of the positive control drug tacrine. Additionally, in the water maze experiment, hyperoside significantly reduced the escape latency of mice, further confirming its effectiveness in enhancing spatial learning and memory functions. The mechanism underlying these effects may be closely associated with the inhibition of acetylcholinesterase (AChE) activity, alleviation of oxidative stress, and protection of cholinergic neuron function (Nam and Lee, 2014). Researchers utilized Amyloid Precursor Protein/Presenilin-1 (APP/PS1) transgenic mice as models for Alzheimer’s disease. The experiment commenced when the mice were 3 months old, providing them with a mixed diet containing 50 mg/kg of hyperoside (high purity > 95%) for 9 months. Through a series of behavioral tests, it was found that preventive treatment with hyperoside significantly enhanced the spatial learning and memory abilities of the mice. Furthermore, additional studies demonstrated that hyperoside inhibits the production of Aβ by significantly reducing the expression and activity of BACE1. Concurrently, by inhibiting the activation of GSK3β, hyperoside reduces the abnormal phosphorylation of Tau protein, as well as the levels of oxidative stress and the expression of inflammatory markers (Chen L. et al., 2021). Hyperoside enhances hippocampal synaptic plasticity by modulating calcium-permeable AMPA receptors (CP-AMPARs) and the upstream Adenylyl Cyclase/Protein Kinase A (AC/PKA) signaling pathway. This mechanism contributes to the amelioration of learning and memory deficits in AD mice induced by Aβ(1–42). Passive avoidance and object recognition experiments further corroborated that hyperoside improved the memory performance of these mice. In mechanistic studies, hyperoside (30 μM) demonstrated its effects by facilitating long-term potentiation (LTP) in the hippocampus. This effect was dependent on Calcium-Permeable AMPA Receptors (CP-AMPARs) and could be inhibited by the CP-AMPAR antagonist IEM-1460. Furthermore, hyperoside promotes the synaptic expression of CP-AMPARs via the AC/PKA signaling pathway (Yi et al., 2022). In the *Caenorhabditis elegans* model, the compound significantly reduced ROS levels induced by Aβ42 and improved both chemotaxis impairment and learning deficits associated with Aβ42 exposure. To further investigate the underlying mechanisms, the researchers established an injury model using Aβ42-treated PC12 cells. JC-1 fluorescent staining results demonstrated that treatment with hyperoside (at concentrations of 10, 20, and 30 μM) significantly restored mitochondrial membrane potential (MMP) and ameliorated mitochondrial dysfunction. Additional mechanistic studies indicated that hyperoside exerts its antioxidant and anti-apoptotic effects by activating the PI3K/Akt/Nrf2/HO-1 signaling pathway. Furthermore, molecular docking studies suggest that the PI3K/Akt pathway may also play a role in the protective effects of hyperoside against Aβ42-induced damage in PC12 cells (Wang et al., 2023b). This study investigates the effects of compound AD, specifically in APP/PS1 double transgenic mice and the HT22 cell line. Hyperoside significantly mitigates Aβ-related neurotoxicity through multiple mechanisms. Its mode of action involves direct binding to Aβ, which inhibits its aggregation and decreases the formation of oligomers and fibrous structures. Additionally, hyperoside markedly reduces Aβ production by inhibiting β-secretase (BACE1) activity. Furthermore, it modulates the calcium signaling pathway between the endoplasmic reticulum and mitochondria, successfully restoring calcium homeostasis and safeguarding mitochondrial function. Hyperoside also stabilizes the mitochondrial membrane potential and inhibits the mitochondrial apoptosis pathway induced by Aβ. Results from animal experiments indicate that intranasal administration of hyperoside (at doses of 20, 40, and 80 mg/kg, administered once every 3 days for 8 weeks) significantly improved cognitive function and motor coordination in APP/PS1 double-transgenic Alzheimer’s disease mice. Analysis of mouse brain tissue revealed that hyperoside not only diminished Aβ plaque deposition in the cortex and hippocampus but also reduced the expression of GFAP, a marker of astrocyte activation (Song et al., 2023).

Hyperoside is a natural compound known for its diverse neuroprotective effects and has garnered significant attention due to its potential in treating neurodegenerative diseases. Epidemiological studies indicate that a diet rich in antioxidant and anti-inflammatory compounds positively influences the prevention of Alzheimer’s disease (Calderaro et al., 2022; Minocha et al., 2022). Consequently, future research should extend beyond laboratory model systems and progressively advance towards clinical applications to systematically assess the effectiveness and feasibility of hyperoside in human subjects.

## 13 Conclusion and future perspectives

Hyperoside is a natural flavonoid compound derived from plants. Due to its diverse biological functions and multi-target regulatory mechanisms, it holds significant potential for the treatment of various diseases. Recent research has demonstrated that hyperoside can effectively modulate biological processes such as cell proliferation, apoptosis, autophagy, and oxidative stress. Notably, hyperoside has exhibited substantial efficacy in therapeutic experiments targeting tumors, including lung cancer and colorectal cancer, as well as non-tumor conditions such as fibrosis and metabolic disorders. However, much of the current research on hyperoside remains at the basic experimental stage, and its mechanisms of action have not yet been thoroughly analyzed. Additionally, the low bioavailability and drug stability of hyperoside pose challenges to its clinical application. Therefore, conducting multi-level and systematic research is essential for advancing the clinical use of hyperoside.

In the realm of basic research, future efforts should concentrate on the comprehensive investigation of the mechanism of action of hyperoside, particularly its functional performance within complex biological environments. Currently, most research emphasizes the direct mechanisms of action of hyperoside on cancer cells, while systematic studies regarding its regulation of the tumor microenvironment are notably scarce. The tumor microenvironment, a critical factor influencing tumor initiation, progression, and treatment resistance, comprises tumor-associated fibroblasts (CAFs), tumor-associated macrophages (TAMs), immune checkpoints (such as PD-L1), and various signaling pathways. Investigating the efficacy of hyperoside’s intervention in these areas may pave the way for novel therapeutic strategies aimed at targeting the tumor microenvironment. For instance, hyperoside may enhance anti-tumor immune responses by modulating the polarization of TAMs or by increasing the sensitivity of tumor cells to the immune system through the inhibition of PD-L1 expression. Integrating multi-omics technologies—such as transcriptomics, metabolomics, and proteomics—to construct a target network of hyperoside within the tumor microenvironment will not only elucidate its intricate mechanism of action but also provide a theoretical foundation for personalized treatment plans. Furthermore, hyperoside’s multi-target properties confer significant potential for combination therapies, where its synergistic effects with traditional chemotherapy, targeted therapy, or immunotherapy could effectively address the limitations inherent in single treatment modalities. By simulating the synergistic effects of hyperoside and other drugs at the cellular and tissue levels through systems biology methods, and employing artificial intelligence-driven drug design tools to optimize the structural derivatives of hyperoside, we can significantly expand its therapeutic applications. For instance, in patients resistant to chemotherapy, hyperoside can be combined with traditional chemotherapy agents such as paclitaxel to markedly enhance drug sensitivity by modulating the NF-κB and TLR4 signaling pathways. A thorough exploration of this synergistic mechanism will not only expedite the clinical application of hyperoside but also provide valuable insights for the development of other natural drugs. The clinical potential of hyperoside is enhanced by their unique multi-targeting properties, which distinguish them from conventional therapies. In contrast to traditional chemotherapeutic or targeted agents, hyperoside demonstrate lower toxicity and possess the ability to modulate tumor cells and their microenvironment, including immune checkpoints and inflammatory pathways. These characteristics position hyperoside as important candidates for combination therapy (e.g., with paclitaxel) aimed at overcoming chemoresistance and improving therapeutic efficacy. However, current research primarily focuses on the preclinical phase and lacks direct comparative studies with existing treatments regarding efficacy and safety. Consequently, high-quality preclinical and clinical studies are essential to critically evaluate these aspects and to investigate their potential as monotherapeutic agents or in combination regimens.

Despite the diverse pharmacological effects of hyperoside, its low bioavailability and drug stability remain significant bottlenecks that hinder its widespread application. To address these challenges, future efforts should concentrate on the development of innovative drug delivery systems that enable precise delivery and dynamic regulation. The application of nanoformulation in drug delivery systems has garnered considerable attention in recent years. Its advantages encompass improved drug solubility, enhanced bioavailability, targeted delivery, and a reduction in toxicity and side effects. Studies have demonstrated that nanotechnology-based hyperoside delivery systems can significantly enhance the drug’s activity. For instance, cell membrane-camouflaged nanocarriers can mimic the properties of natural cell membranes, effectively preventing premature clearance of the drug in the bloodstream and significantly enhancing the blood concentration of hyperoside. Additionally, mitochondria-targeted delivery systems can transport hyperoside directly to the cell mitochondria in response to the mitochondrial membrane potential, further augmenting its efficacy. Regarding release strategies, the integration of microenvironment-responsive technologies, such as pH-sensitive or temperature-controlled release mechanisms, can facilitate “on-demand” release, thereby minimizing systemic toxicity and maximizing therapeutic effectiveness. To achieve this objective, researchers have developed composite nanoparticles by combining hyperoside with zein and pectin, as well as a trio of hyperoside-loaded zein-tea polyphenols-pectin metacomposite nanoparticles, aimed at slowing the release rate of hyperoside. This novel nanoparticle system significantly enhances the bioavailability of hyperoside (Wang et al., 2020; Wang et al., 2021). Nanotechnology-based hyperoside preparations are anticipated to enable precise treatment of various diseases. This includes enhancing the anti-cancer effects through targeted delivery technology, minimizing side effects on healthy tissues, and further improving anti-inflammatory and antioxidant medicinal efficacy. Additionally, the potential applications of hyperoside nanoformulation in combination therapies warrant in-depth exploration, offering new possibilities for future drug development and clinical transformation. The potential of hyperoside in immunometabolic remodeling warrants further investigation. Recent advancements in metabolomics have provided a novel perspective on the interplay between energy metabolism and immune function. Hyperoside may play a role in tumor metabolic reprogramming and the establishment of an immunosuppressive microenvironment by modulating oxidative phosphorylation and lipid metabolism. Future research should integrate metabolomics and immunomics technologies to examine the regulatory effects of hyperoside on key metabolic enzymes and metabolites, thereby elucidating its role in immune-related diseases, including autoimmune disorders and organ transplant rejection. This application offers a scientific basis for further exploration. Additionally, hyperoside may enhance immune cell activity by influencing mitochondrial function, thus presenting new strategies for modulating immune responses in chronic inflammatory diseases such as diabetes and non-alcoholic fatty liver disease.

Hyperoside, a flavonoid glycoside, is widely distributed in nature and can be extracted from various plants, including *H. perforatum*, Crataegus pinnatifida, and Acanthopanax senticosus (du et al., 2015; Ning et al., 2015; Xu et al., 2016). Traditional methods, such as ethanol reflux extraction, are commonly employed; however, these methods are increasingly complemented by advanced techniques, including enzyme-assisted extraction, aqueous two-phase extraction, and ionic liquid-assisted extraction (Yao et al., 2013; Enbo et al., 2016; Lei et al., 2018; Shi et al., 2019). From an industrial standpoint, the current production of hyperoside predominantly relies on natural extraction methods, which are constrained by raw material availability, low yields, and high extraction costs. To facilitate large-scale production, the incorporation of synthetic biology technology may represent a significant breakthrough. Optimizing microbial production systems through metabolic engineering, utilizing organisms such as *Escherichia coli* or yeast, could not only substantially increase the yield of hyperoside but also promote more environmentally friendly and low-pollution industrial production. The integration of deep eutectic solvent-assisted extraction technology or microwave-assisted separation technology is anticipated to further reduce production costs, enhance extraction purity, and provide more reliable technical support for the large-scale application of hyperoside. Despite the significant progress made in the basic research of hyperoside, its transition from laboratory studies to clinical applications continues to face numerous challenges. These challenges encompass not only the comprehensive elucidation of the mechanism of action but also the optimization of delivery technologies and the assessment of multidimensional toxicology. In the future, high-quality clinical studies will be essential to verify the efficacy of hyperoside and its long-term safety. Furthermore, the incorporation of interdisciplinary technologies, such as artificial intelligence-assisted drug optimization, multi-omics integrated analysis, and the application of synthetic biology, is expected to drive the further development of hyperoside.

Hyperoside possesses multifunctional and multi-target properties, indicating significant potential for application in tumor treatment and chronic disease management. However, the transition from laboratory research to clinical application presents numerous challenges, including the need for in-depth analysis of its molecular mechanisms, optimization of delivery strategies, toxicological assessments, and long-term safety evaluations. With the rapid advancement of precision medicine and multidisciplinary technologies, hyperoside is poised not only for clinical use as an independent drug but also as an essential component in combination therapies. Ongoing research and innovative practices will invigorate the application of hyperoside in disease treatment and health management while providing crucial guidance and reference for the development of natural compound-based drugs.
